# PDE3B as a multi-organ therapeutic node in the gut–liver axis of metabolic dysfunction-associated steatohepatitis

**DOI:** 10.1038/s12276-026-01811-2

**Published:** 2026-09-01

**Authors:** Yu Seol Lee, Chang-Yun Jeong, Youn Wook Chung, Yoseob Lee, Da Hyun Lee, Ji Yun Bang, Da Ye Kim, Haram Lee, Soohwan Oh, Jinhyuk Bhin, Ji-Hwan Ryu, June-Yong Lee, Soo Han Bae

**Affiliations:** 1 Department of Biomedical Sciences, Yonsei University College of Medicine, Seoul, Republic of Korea; 2 Graduate School of Medical Science, Brain Korea 21 Project, Yonsei University College of Medicine, Seoul, Republic of Korea; 3 Department of Microbiology, Yonsei University College of Medicine, Seoul, Republic of Korea; 4 Center for Microbiome-Body-Brain Physiology, The Institute for Basic Science (IBS), Gwangju Institute of Science and Technology (GIST), Gwangju, South Korea; 5 College of Pharmacy, Korea University, Sejong, Republic of Korea; 6 Department of Biomedical System Informatics, Gangnam Severance Hospital, Yonsei University College of Medicine, Seoul, Republic of Korea; 7 Institute of Immunology and Immunological Diseases, Yonsei University College of Medicine, Seoul, Republic of Korea

**Keywords:** Mechanisms of disease, Metabolic disorders

## Abstract

Metabolic dysfunction-associated steatohepatitis (MASH) involves pathological crosstalk within the gut–liver axis, in which intestinal barrier dysfunction facilitates the translocation of microbial and dietary products and aggravates hepatic inflammation and fibrosis. Although phosphodiesterase 3B (PDE3B) is known to regulate metabolic and inflammatory processes, its role in gut–liver communication remains unclear. Here, we demonstrate that genetic ablation of PDE3B and pharmacological PDE3 inhibition using cilostamide significantly improve hepatic and intestinal features of MASH in diet-induced mouse models. Specifically, PDE3B loss and pharmacological PDE3 inhibition preserve intestinal barrier integrity by restoring tight junction protein expression and reducing intestinal permeability, accompanied by attenuated hepatic inflammatory and fibrotic responses. Mechanistically, loss of PDE3B suppresses nuclear factor-κB-driven inflammatory signaling through activation of a cAMP-protein kinase A-dependent pathway, as reflected by increased phosphorylation of cAMP response element-binding protein. Notably, ileal PDE3B protein declines earlier than hepatic PDE3B during MASH progression, supporting early intestinal engagement during diet-induced metabolic stress. These findings support PDE3B/PDE3 signaling as a therapeutically tractable multi-organ regulatory node of gut–liver axis pathology in MASH and identify pharmacological PDE3 inhibition as a viable strategy to mitigate hepatic injury while preserving intestinal barrier-associated function.

**Figure:**
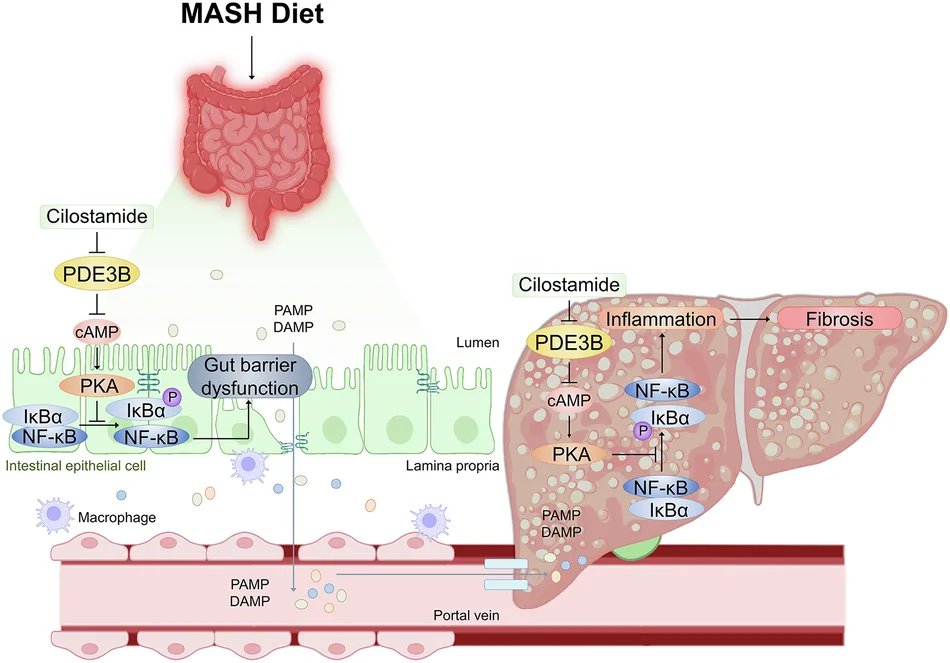

## PDE3B inhibition restores gut–liver function in MASH

Metabolic dysfunction-associated steatohepatitis (MASH) is a major global health issue, affecting about 30% of the population. This study explores the role of phosphodiesterase 3B (PDE3B) in MASH, focussing on its impact on liver inflammation, fibrosis, and intestinal barrier integrity. Using a diet-induced mouse model, researchers used genetic ablation and pharmacological inhibition to assess the role of PDE3B in gut–liver axis pathology. They found that PDE3B deficiency reduced liver fibrosis and inflammation, decreased hepatic lipid accumulation, and preserved intestinal barrier function. These effects were linked to the modulation of nuclear factor-κB signaling via the cAMP–protein kinase A (PKA) pathway. The study suggests that targeting PDE3B could offer a multi-organ therapeutic strategy for MASH, with cilostamide showing potential as a PDE3 inhibitor. Future research should explore tissue-specific PDE3B roles and further validate these findings in clinical settings. DAMP, damage-associated molecular pattern; PAMP, pathogen-associated molecular pattern.

This summary was initially drafted using artificial intelligence, then revised and fact-checked by the author.

## Introduction

Metabolic dysfunction-associated steatotic liver disease (MASLD) affects ~30% of the global population, representing a growing public health concern^1,2^. MASLD encompasses a spectrum ranging from benign hepatic steatosis to metabolic dysfunction-associated steatohepatitis (MASH), an advanced stage characterized by excessive hepatic fat accumulation, chronic inflammation, and progressive fibrosis^3^. If left untreated, MASH can progress to severe hepatic complications, including cirrhosis and hepatocellular carcinoma, often requiring liver transplantation as the only definitive treatment^4^. Beyond liver-specific pathology, MASH is also associated with systemic metabolic disturbances, including compromised intestinal barrier integrity, which highlights the importance of gut–liver axis dysfunction in disease progression^5–8^. These interconnected pathologies complicate disease management and underscore the urgent need for therapeutic strategies that can simultaneously target multiple organs.

The pathogenesis of MASH is multifactorial and involves complex interorgan signaling, particularly between the liver and intestine^9–11^. Growing evidence highlights the gut–liver axis as a critical contributor to MASH development. Specifically, disruption of intestinal epithelial tight junction, such as those formed by zonula occludens-1 (ZO-1), occludin (OCLN), and claudins (CLDNs), increases intestinal permeability, allowing translocation of microbial and dietary components (for example, lipopolysaccharide (LPS)) into the portal circulation^12^. This can engage hepatic and intestinal nuclear factor (NF)-κB signaling, amplifying systemic inflammation and metabolic dysregulation^13,14^. However, the regulatory mechanisms that coordinate NF-κB activation across the gut–liver axis remain poorly defined. Given the central role of NF-κB in MASH pathogenesis, identifying key modulators that simultaneously influence both hepatic and intestinal inflammatory signaling is crucial for the development of multi-organ therapeutic strategies.

Phosphodiesterase 3B (PDE3B) is a cAMP-degrading enzyme implicated in regulating metabolic and inflammatory processes^15,16^. In metabolic disease models, PDE3B promotes insulin resistance, inhibits lipolysis, and enhances fat storage in adipose tissues. Notably, PDE3B-deficient mice exhibit reduced adipose inflammation, decreased macrophage infiltration, and suppressed activation of inflammasomes such as NLR family pyrin domain containing 3 (NLRP3) (refs. ^17,18^). Furthermore, in the cardiovascular system, disruption of PDE3B has been shown to reduce mitochondrial oxidative stress and increase anti-apoptotic proteins such as Bcl-2 (ref. ^19^). As a cAMP-specific phosphodiesterase, PDE3B is well positioned to modulate cAMP-protein kinase A (PKA) signaling, a pathway whose downstream cAMP response element-binding protein (CREB)-dependent transcriptional responses have been implicated in epithelial barrier homeostasis and inflammatory regulation^20–22^. However, whether PDE3B coordinates hepatic inflammation, intestinal epithelial barrier dysfunction, and immune-cell responses within the gut–liver axis remains unknown. Given this gap, we hypothesized that PDE3B may act as a multi-organ regulatory node that connects intestinal barrier disruption with hepatic inflammatory signals during MASH.

In this study, we investigated the expression and functional role of PDE3B in hepatic steatosis, inflammation, fibrosis, and intestinal barrier integrity using a well-established diet-induced MASH mouse model. This dietary model was selected because it recapitulates key hepatic features of MASH and is accompanied by measurable intestinal barrier dysfunction. By combining genetic ablation and pharmacological inhibition approaches, we sought to define the contribution of PDE3B to gut–liver axis pathology and to evaluate its potential as a multi-organ therapeutic target in MASH.

## Material and methods

### Animals

Male C57BL/6J mice were obtained from Japan SLC, Inc., and PDE3B wild-type and knockout mice were generated as described previously^19,23^. All mice were maintained in a temperature-controlled facility (23 ± 2 °C) with ad libitum access to water and standard chow under a 12 h light-dark cycle. Animal health, including body weight, behavior, and responses to external stimuli, was monitored daily. All experimental procedures were approved by the Animal Care and Use Committee of Yonsei University College of Medicine, and measures were taken to minimize animal distress (approval numbers: 2020-0274, 2022-0345, and 2023-0164).

All animal experiments were conducted and reported in accordance with the ARRIVE 2.0 (Animal Research: Reporting of In Vivo Experiments) guidelines^24^. The study design, experimental procedures, and data analysis adhered to these guidelines to ensure transparency, reproducibility, and ethical considerations in animal research.

### Statistical analysis

All data are presented as mean ± SEM. Two-group comparisons were conducted using Student’s *t* test, whereas comparisons among multiple groups were performed with one-way analysis of variance followed by Tukey’s post hoc test. GraphPad Prism (v10.2.2) was used for all statistical analyses. Details of software used are provided in Supplementary Table 5.

For more details on the materials and methods used, refer to the Supplementary information.

## Results

### Hepatic PDE3B expression is post-transcriptionally reduced in MASH

**Fig. 1:**
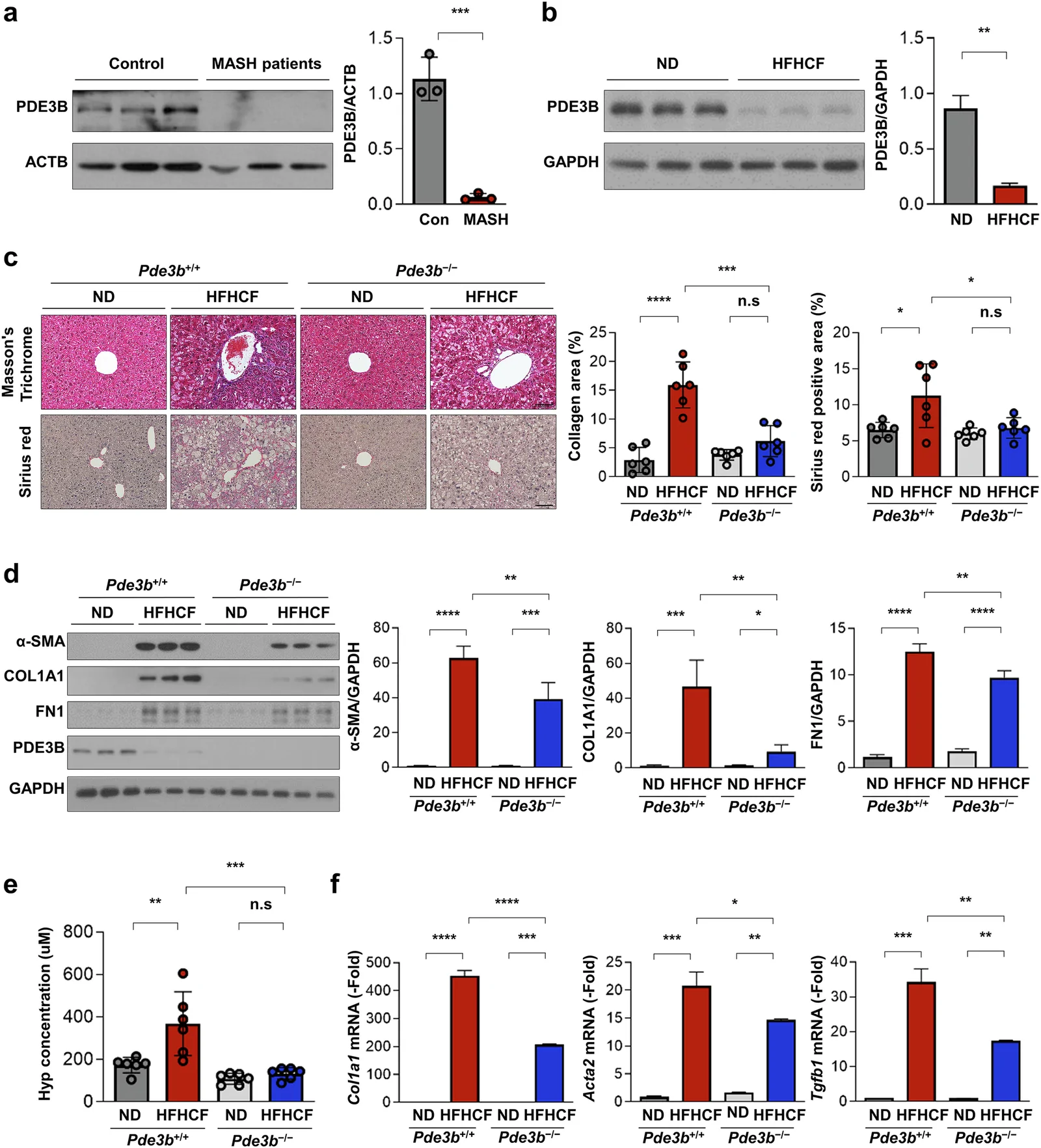
PDE3B deficiency alleviates liver fibrosis in MASH. **a** Western blot analysis of liver biopsies from normal individuals and patients with metabolic dysfunction-associated steatohepatitis (MASH) and quantification of phosphodiesterase 3B (PDE3B) normalized to ACTB (*n* = 3 per group). **b** Western blot analysis of liver pooling sample from 8 weeks HFHCF mice model and quantification of PDE3B normalized to GAPDH (*n* = 5 per group). **c–f** *Pde3b*^+/+^ and *Pde3b*^−/−^ mice were fed a normal diet (ND) or HFHCF diet for 8 weeks (*n* = 6 per group). **c** Representative images of Masson’s trichrome (top) and Sirius red (bottom) staining of liver sections with quantification of positive area per field. Scale bar, 100 µm. **d** Western blot analysis of liver tissues with antibodies against alpha-smooth muscle actin (α-SMA), COL1A1, FN1, PDE3B, and GAPDH as a loading control, and densitometric analysis of them normalized to GAPDH (*n* ≥ 5 per group). **e** Hepatic hydroxyproline (Hyp) content normalized to liver tissue weight. **f** Relative *Col1a1*, *Acta2*, and *Tgfb1* mRNA levels in liver tissue. Data are presented as mean ± SEM. *P < 0.0332; **P < 0.0021; ***P < 0.0002; and ****P < 0.0001. n.s=non-significant.

To investigate the role of PDE3B in MASH, we first examined its expression in liver samples from both patients and mice with MASH. PDE3B protein levels were significantly reduced in samples of patients with MASH, whereas mRNA levels remained unchanged (Fig. 1a and Supplementary Fig. 1a). Public transcriptomic data (Gene Expression Omnibus (GEO) dataset GSE48452) further support this protein–mRNA dissociation across normal, steatotic, and MASH liver samples (Supplementary Fig. 1b). Similarly, in various diet-induced MASH mouse models, hepatic PDE3B protein expression was uniformly reduced despite preserved or even increased *Pde3b* mRNA levels (Fig. 1b and Supplementary Fig. 1c–i). To probe the mechanism underlying this discordance, we modeled MASH-like stress in Caco-2 cells using MASH-like cocktail (MLC; 500 μM palmitate, 1% lipid mixture, 50 μM fructose, and 1 μg/ml LPS). Under these conditions, PDE3B protein decreased without a concordant reduction in *Pde3b* transcripts (Supplementary Fig. 1j–l). Pharmacological inhibition indicated a lysosome-dependent autophagy contribution to PDE3B loss, whereas proteasome blockade afforded only limited rescue (Supplementary Fig. 1m–o). Collectively, these data indicate that PDE3B is reduced in MASH predominantly at the protein level and suggest that lysosome-dependent autophagic turnover can contribute to PDE3B loss under MASH-like stress, rather than transcriptional suppression.

### PDE3B deficiency attenuates liver fibrosis in mice with MASH

Next, to assess the functional impact of PDE3B deficiency on liver fibrosis in MASH, we validated *Pde3b*^−/−^ mice (Supplementary Fig. 2a,b). Although the high-fat, high-cholesterol diet supplemented with a fructose water solution (HFHCF) regimen effectively induced MASH, there were no statistically significant differences in serum alanine aminotransferase (ALT) levels or liver-to-body-weight ratio between *Pde3b*^+/+^ and *Pde3b*^−/−^ mice (Supplementary Fig. 2c–e). Notably, despite comparable ALT and liver-to-body-weight ratios, *Pde3b*^−/−^ mice displayed markedly reduced fibrosis compared with their *Pde3b*^+/+^ counterparts, as evidenced by Masson’s trichrome and Sirius red staining (Fig. 1c). Correspondingly, the protein levels of fibrosis-related markers — including alpha-smooth muscle actin (α-SMA), COL1A1, and FN1 — were significantly lower in *Pde3b*^−/−^ mice (Fig. 1d), and hydroxyproline content, reflecting collagen deposition, was also reduced in *Pde3b*^−/−^ (Fig. 1e). Furthermore, fibrosis-related genes such as *Col1a1*, *Acta2* (encoding α-SMA), and *Tgfb1* were upregulated in HFHCF-fed *Pde3b*^+/+^ mice, but showed attenuated expression in *Pde3b*^−/−^ mice (Fig. 1f). Together, the data uncover an underappreciated profibrogenic role for PDE3B in diet-induced MASH, highlighting its pathogenic relevance beyond metabolic regulation.

### PDE3B deficiency attenuates NF-κB-associated hepatic inflammation and immune-cell infiltration in MASH

**Fig. 2:**
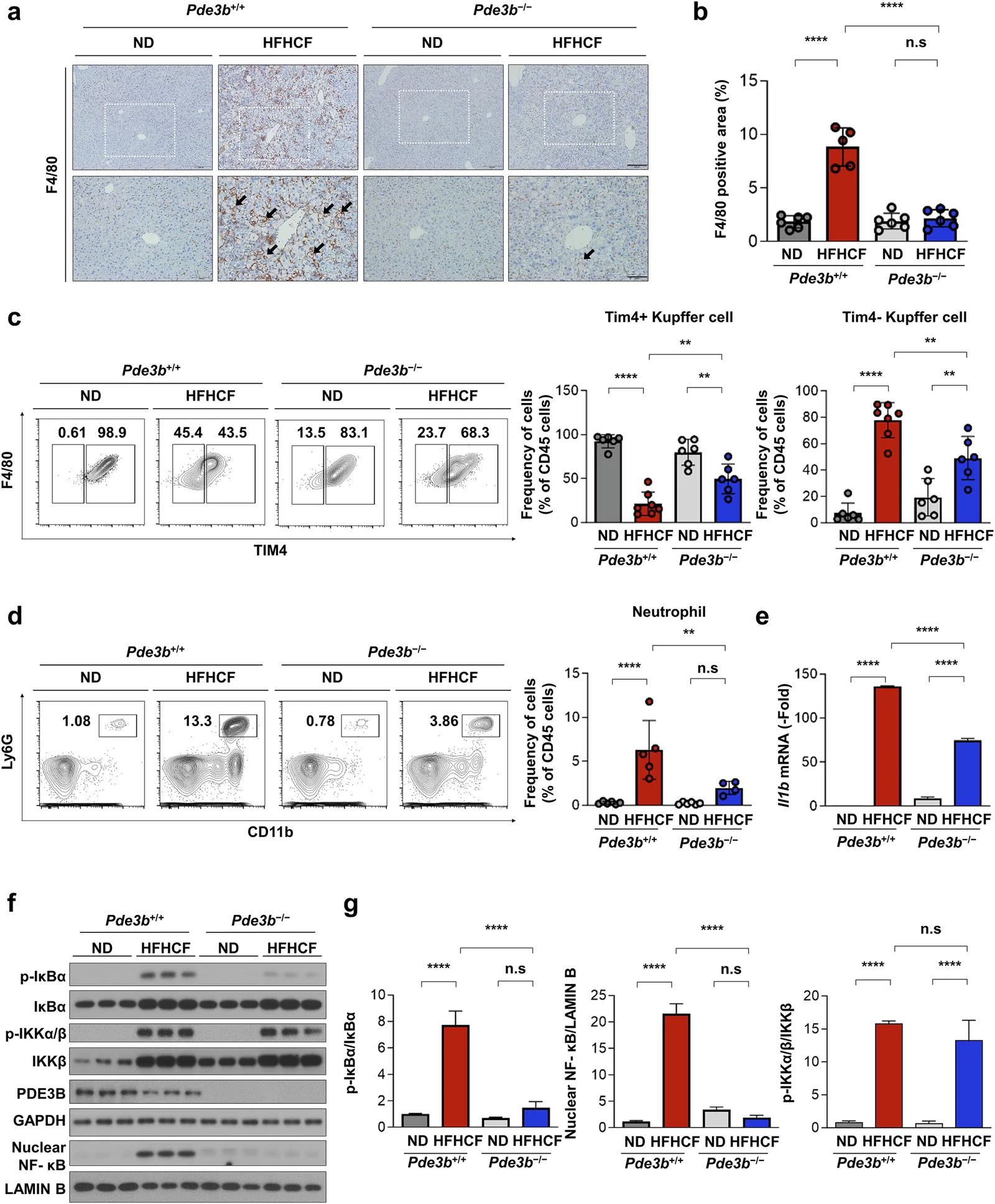
PDE3B deficiency ameliorates hepatic inflammation by reducing NF-κB activation and macrophage infiltration. *Pde3b*^+/+^ and *Pde3b*^−/−^ mice were fed a normal diet (ND) or HFHCF diet for 8 weeks. **a**, **b** Representative images of liver sections stained with the F4/80 antibody. Scale bars, 200 µm (top) and 100 µm (bottom). Quantification of the F4/80-positive areas per field. *n* = 6 per group. **c** Representative flow cytometry plots and frequencies of Tim4^+^-resident Kupffer cells and Tim4^−^ infiltrating macrophages among CD45^+^ cells in the liver. *n* = 6 or 7 per group. **d** Representative flow cytometry plots and frequencies of neutrophils among CD45^+^ cells in the liver. *n* = 6 or 4 per group. **e** Relative *Il1b* mRNA levels in liver tissue. *n* ≥ 5 per group. **f** Western blot analysis of liver tissues with antibodies against p-IκBα, IκBα, p-IKKα/β, IKKβ, phosphodiesterase 3B (PDE3B), and GAPDH as a loading control, nuclear NF-κB and LAMIN B as loading control of nuclear extracts. **g** Densitometric analysis of p-IκBα normalized to IκBα, nuclear NF-κB normalized to LAMIN B, and p-IKKα/β normalized to IKKβ (*n* ≥ 5 per group). Data are presented as mean ± SEM. **P < 0.0021 and ****P < 0.0001. n.s=non-significant.

We next asked whether loss of PDE3B restrains hepatic inflammation, focusing on macrophage dynamics in HFHCF‑fed mice. Immunohistochemical staining for F4/80 revealed a significant increase in hepatic macrophage infiltration in *Pde3b*^+/+^ mice, which was notably reduced in *Pde3b*^−/−^ mice (Fig. 2a, b).

Previous lineage-tracing studies have shown that MASH depletes resident Tim4^+^ Kupffer cells, thereby promoting the recruitment of Tim4^−^ monocyte-derived Kupffer cells with a heightened inflammatory potential^25,26^. To determine whether PDE3B affects Kupffer cell subpopulations, we performed flow cytometric analysis to distinguish between resident (Tim4^+^) and infiltrating (Tim4^−^) macrophages. In *Pde3b*^+/+^ MASH livers, resident Tim4^+^ Kupffer cells were depleted, whereas inflammatory Tim4^−^ monocyte-derived cells were expanded. By contrast, *Pde3b*^−/−^ mice showed a less pronounced loss of Tim4^+^ Kupffer cells and reduced infiltration of Tim4^−^ macrophages (Fig. 2c and Supplementary Fig. 3a).

Consistent with these findings, the neutrophil infiltration commonly observed in MASH livers^27,28^ was markedly attenuated in *Pde3b*^−/−^ mice (Fig. 2d). To determine whether these immune-cell changes resulted from cell-intrinsic effects of PDE3B deletion, we analyzed a publicly available mouse liver single-cell RNA sequencing dataset (GSE192740). This analysis showed that PDE3B expression is primarily localized in hepatocytes, with minimal expression in Kupffer cells, monocyte-derived cells, and neutrophils (Supplementary Fig. 3b,c). These findings suggest that altered immune-cell recruitment in *Pde3b*^−/−^ mice is unlikely to be driven primarily by immune-cell-intrinsic PDE3B loss, but may instead reflect changes in non-immune-cell compartments, extrahepatic inputs, or both.

The altered immune-cell landscape was accompanied by decreased hepatic expression of *Il1b* and other pro-inflammatory genes in *Pde3b*^−/−^ mice (Fig. 2e and Supplementary Fig. 3d,e). At the signaling level, the phosphorylation of IκBα and nuclear translocation of NF-κB, hallmarks of canonical inflammatory activation, were reduced in the liver of *Pde3b*^−/−^ mice (Fig. 2f, g).

Collectively, these results demonstrate that PDE3B deficiency suppresses hepatic inflammation in MASH by modulating Kupffer cell dynamics, reducing neutrophil infiltration, and attenuating NF-κB**-**mediated signaling.

### Cilostamide-mediated PDE3 inhibition alleviates MASH features

**Fig. 3:**
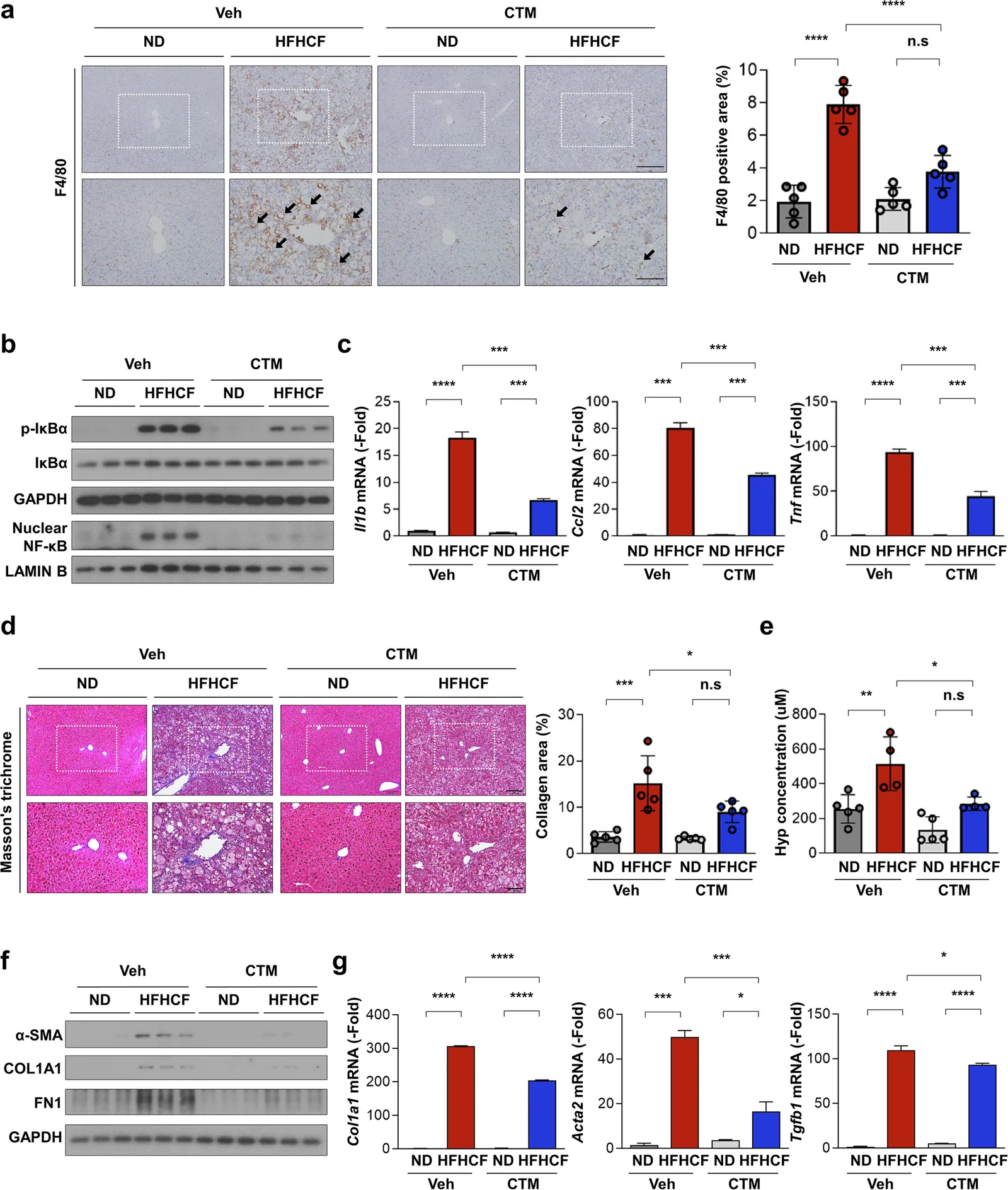
Pharmacological inhibition of PDE3 reduces hepatic inflammation and fibrosis in MASH. C57BL/6J mice were fed a normal diet (ND) or the HFHCF diet for 4 weeks, followed by treatment with either vehicle (Veh) or cilostamide (CTM; 3 mg/kg) for an additional 4 weeks while continuing the respective diets (*n* = 5 per group). **a** Representative images of liver sections stained with the F4/80 antibody. Scale bar, 200 μm (top) and 100 μm (bottom). Quantification of the F4/80-positive areas per field. **b** Western blot analysis of liver tissues with antibodies against p-IκBα, IκBα, GAPDH as a loading control, nuclear NF-κB and LAMIN B as loading control of nuclear extracts. **c** Relative *Il1b*, *Ccl2*, and *Tnf* mRNA levels in liver tissue. **d** Representative Masson’s trichrome staining of liver sections. Scale bars, 200 µm (top) and 100 µm (bottom). **e** Hepatic hydroxyproline (Hyp) content normalized to the tissue weight. **f** Western blot analysis of liver tissues with antibodies against α-SMA, COL1A1, FN1, and GAPDH as a loading control. **g** Relative *Col1a1*, *Acta2*, and *Tgfb1* mRNA levels in liver tissue. Data are presented as mean ± SEM. *P < 0.0332; **P < 0.0021; ***P < 0.0002; and ****P < 0.0001. n.s=non-significant. MASH, metabolic dysfunction-associated steatohepatitis; PDE3B, phosphodiesterase 3B.

To evaluate the therapeutic potential of pharmacological PDE3 inhibition in MASH management, we orally administered cilostamide, a selective PDE3 inhibitor, to MASH-model mice from week 4 to week 8 of HFHCF feeding. Cilostamide treatment significantly reduced hepatic inflammation and fibrosis. Immunohistochemical analysis for F4/80 revealed decreased hepatic macrophage infiltration in cilostamide-treated mice, accompanied by lower levels of phosphorylated IκBα (Fig. 3a, b), and reduced expression of inflammatory genes, including *Il1b*, *Ccl2*, and *Tnf* (Fig. 3c). Consistent with these findings, Masson’s trichrome staining and hydroxyproline quantification demonstrated a marked reduction in liver fibrosis. Fibrosis-associated proteins and transcripts similarly declined after cilostamide (Fig. 3d–g), consolidating the antifibrotic profile.

### Genetic and pharmacological targeting PDE3B reduces hepatic lipid accumulation in MASH

Given the critical role of lipid metabolism in MASH pathogenesis, we next examined hepatic lipid accumulation in *Pde3b*^−/−^ mice under MASH-inducing conditions. Histological analysis revealed substantial lipid accumulation in *Pde3b*^+/+^ mice, which was significantly attenuated in *Pde3b*^−/−^ mice (Supplementary Fig. 4a,b). Hepatic triglyceride content was also reduced in *Pde3b*^−/−^ mice compared with *Pde3b*^+/+^ controls (Supplementary Fig. 4c). These improvements were accompanied by alterations in the expression of lipid metabolism-related genes. In *Pde3b*^+/+^ MASH mice, genes associated with lipid storage and uptake (*Plin2*, *Dgat1*, and *Slc27a4*) were markedly upregulated, but this induction was blunted in *Pde3b*^−/−^ mice. Conversely, genes involved in fatty acid oxidation (*Cpt1a*, *Aco1*), which were suppressed in *Pde3b*^+/+^ MASH mice, were partially preserved in *Pde3b*^−/−^ mice (Supplementary Fig. 4d).

Cilostamide treatment produced similar improvements in hepatic lipid metabolism. Histological analysis indicated reduced hepatic steatosis in cilostamide-treated mice, along with decreased liver triglyceride levels (Supplementary Fig. 4e–g). Gene expression patterns mirrored those observed in *Pde3b*^−/−^ mice, with decreased expression of *Plin2*, *Dgat1*, and *Slc27a4* and preserved expression of *Cpt1a* and *Aco1* (Supplementary Fig. 4h).

To explore whether the reduction in hepatic lipid accumulation was accompanied by changes in systemic lipid availability, we measured serum levels of non-esterified fatty acids at various time points. At 2 weeks of HFHCF feeding, serum non-esterified fatty acid levels were significantly increased in *Pde3b*^+/+^ mice, whereas this increase was blunted in *Pde3b*^−/−^ mice (Supplementary Fig. 4i). By contrast, no significant differences were observed at later time points (4 and 8 weeks; Supplementary Fig. 4j,k), indicating that PDE3B deficiency is associated with an early and transient alteration in circulating lipid availability during HFHCF-induced metabolic stress.

Collectively, these findings indicate that genetic PDE3B deficiency and pharmacological PDE3 inhibition reduce hepatic lipid accumulation in MASH, accompanied by coordinated changes in hepatic lipid metabolism-related genes and early systemic lipid availability. These observations further support PDE3B as a multi-organ regulatory node that links hepatic metabolic remodeling with broader systemic responses during MASH.

### PDE3B deficiency preserves intestinal barrier integrity and mitigates gut–liver axis dysfunction in MASH

**Fig. 4:**
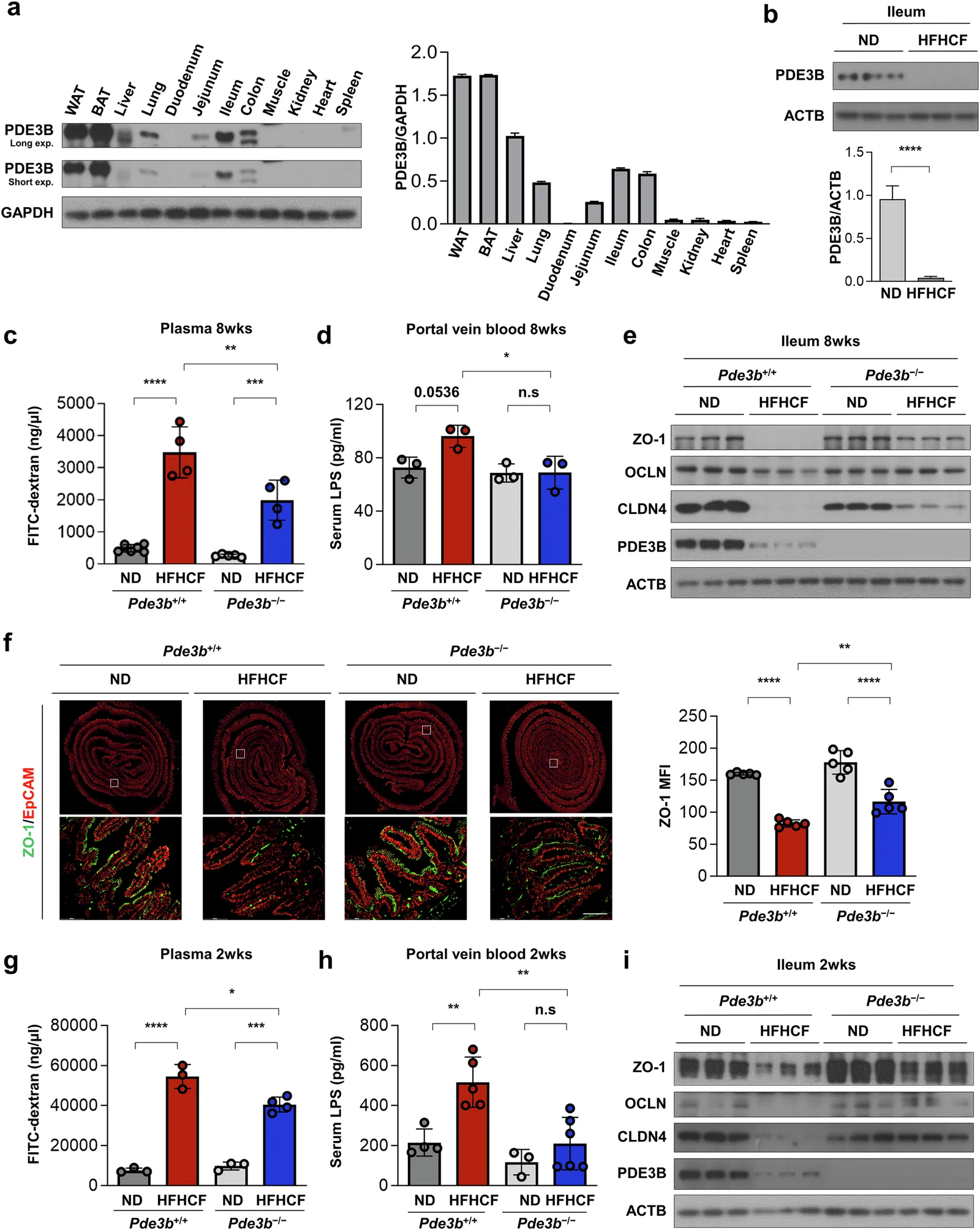
PDE3B deficiency preserves intestinal barrier integrity during MASH pathogenesis. **a** Western blot analysis of multiple tissues from C57BL/6J mice and quantification of phosphodiesterase 3B (PDE3B) normalized to GAPDH. **b** Western blot analysis of ileum pooling sample from 8 weeks HFHCF mice model and quantification of PDE3B normalized to ACTB (*n* = 5 per group). **c**–**f** *Pde3b*^+/+^ and *Pde3b*^−/−^ mice were fed a normal diet (ND) or HFHCF diet for 8 weeks. **c** Plasma fluorescein isothiocyanate (FITC)-dextran fluorescence as a measure of intestinal permeability (6 ≥ *n* ≥ 4 per group). **d** Serum lipopolysaccharide (LPS) levels in portal vein blood (*n* = 3 per group). **e** Western blot analysis of ileum tissues with antibodies against ZO-1, OCLN, CLDN4, PDE3B, and ACTB as a loading control (*n* ≥ 5 per group). **f** Immunofluorescence staining of ileum tissues for ZO-1 and epithelial cell adhesion molecule (EpCAM). Quantification of ZO-1-positive areas. **g**–**i** *Pde3b*^+/+^ and *Pde3b*^−/−^ mice were fed an ND or HFHCF diet for 2 weeks (6 ≥ *n* ≥ 3 per group). **g** Plasma FITC-dextran fluorescence as a measure of intestinal permeability. **h** Serum LPS levels in portal vein blood. **i** Western blot analysis of ileum tissues with antibodies against ZO-1, OCLN, CLDN4, PDE3B, and ACTB as a loading control. Data are presented as mean ± SEM. *P < 0.0332; **P < 0.0021; ***P < 0.0002; and ****P < 0.0001. n.s=non-significant. ACTB, β-actin; BAT, brown adipose tissue; MASH, metabolic dysfunction-associated steatohepatitis; MFI, mean fluorescence intensity; OCLN, occludin; WAT, white adipose tissue; ZO-1, zonula occludens-1.

To investigate the role of PDE3B in extrahepatic metabolic organs relevant to MASH, we assessed its expression across various peripheral tissues. PDE3B was abundantly expressed throughout the intestine, with particularly high levels in distal small intestine and adipose tissue (Fig. 4a). Notably, MASH induction selectively lowered ileal PDE3B protein (Fig. 4b and Supplementary Fig. 5a,b), mirroring hepatic downregulation (Fig. 1b). By contrast, neither mRNA nor protein levels of PDE3B were significantly altered in adipose tissue (Supplementary Fig. 5c,d), suggesting a limited role for this tissue in mediating the observed phenotypes.

Previous studies have linked MASLD and MASH to increased intestinal permeability, primarily due to disruption of epithelial tight junctions. This compromised barrier allows translocation of bacterial endotoxins such as LPS, exacerbating hepatic lipid accumulation and inflammation^13,29,30^. Given prior reports linking MASLD and MASH to increased intestinal permeability and endotoxin translocation, we next evaluated whether PDE3B deficiency influences barrier integrity and the associated hepatic inflammatory response.

In vivo FITC-dextran permeability assays revealed a pronounced increase in intestinal permeability in *Pde3b*^+/+^ MASH mice, whereas *Pde3b*^−/−^ mice exhibited significantly reduced FITC-dextran levels, indicating preserved barrier function (Fig. 4c). In parallel, portal vein LPS concentrations were significantly lower in *Pde3b*^−/−^ mice compared with *Pde3b*^+/+^ controls (Fig. 4d). At the molecular level, MASH-induced reduction in key tight junction proteins was attenuated in *Pde3b*^−/−^ mice (Fig. 4e). Immunofluorescence analysis further confirmed enhanced ZO-1 localization in *Pde3b*^−/−^ ileal epithelium, indicating preserved tight junction integrity (Fig. 4f).

Strikingly, as early as 2 weeks into HFHCF feeding — before overt hepatic fibrosis and without a detectable reduction in hepatic PDE3B protein (Supplementary Fig. 6a–e) — intestinal permeability and portal LPS levels were already markedly reduced in *Pde3b*^−/−^ mice (Fig. 4g, h). At this early stage, ileal PDE3B expression was already reduced in response to HFHCF, and tight junction protein expression remained higher in *Pde3b*^−/−^ mice (Fig. 4i). In addition, levels of phosphorylated IKKα/β and IκBα were significantly lower in the livers of *Pde3b*^−/−^ mice at the 2-week time point, indicating early suppression of NF-κB signaling (Supplementary Fig. 6a, b). Notably, in contrast to the 8-week model in which only p-IκBα was reduced without changes in p-IKKα/β (Fig. 2f, g), these results suggest a time-dependent modulation of the NF-κB pathway by PDE3B deficiency during MASH progression.

Given the involvement of lipid metabolism in MASH, we also assessed serum lipid profiles. At 2 weeks, serum total cholesterol (TCHO) and low-density lipoprotein (LDL) levels were significantly reduced in *Pde3b*^−/−^ mice, whereas high-density lipoprotein (HDL) levels remained unchanged (Supplementary Fig. 6f–h). These changes indicate that PDE3B deficiency is accompanied by early changes in systemic lipid profiles during HFHCF-induced metabolic stress.

Collectively, these findings indicate that ileal PDE3B is downregulated early during HFHCF feeding and that PDE3B deficiency preserves intestinal barrier integrity, reduces portal endotoxin exposure, and attenuates hepatic inflammatory signaling during MASH progression. These results position PDE3B as a multi-organ regulatory node that participates in coordinated changes across intestinal barrier function and hepatic inflammatory-fibrotic outputs in diet-induced MASH.

### PDE3B deficiency attenuates NF-κB-associated intestinal epithelial responses during early MASH

**Fig. 5:**
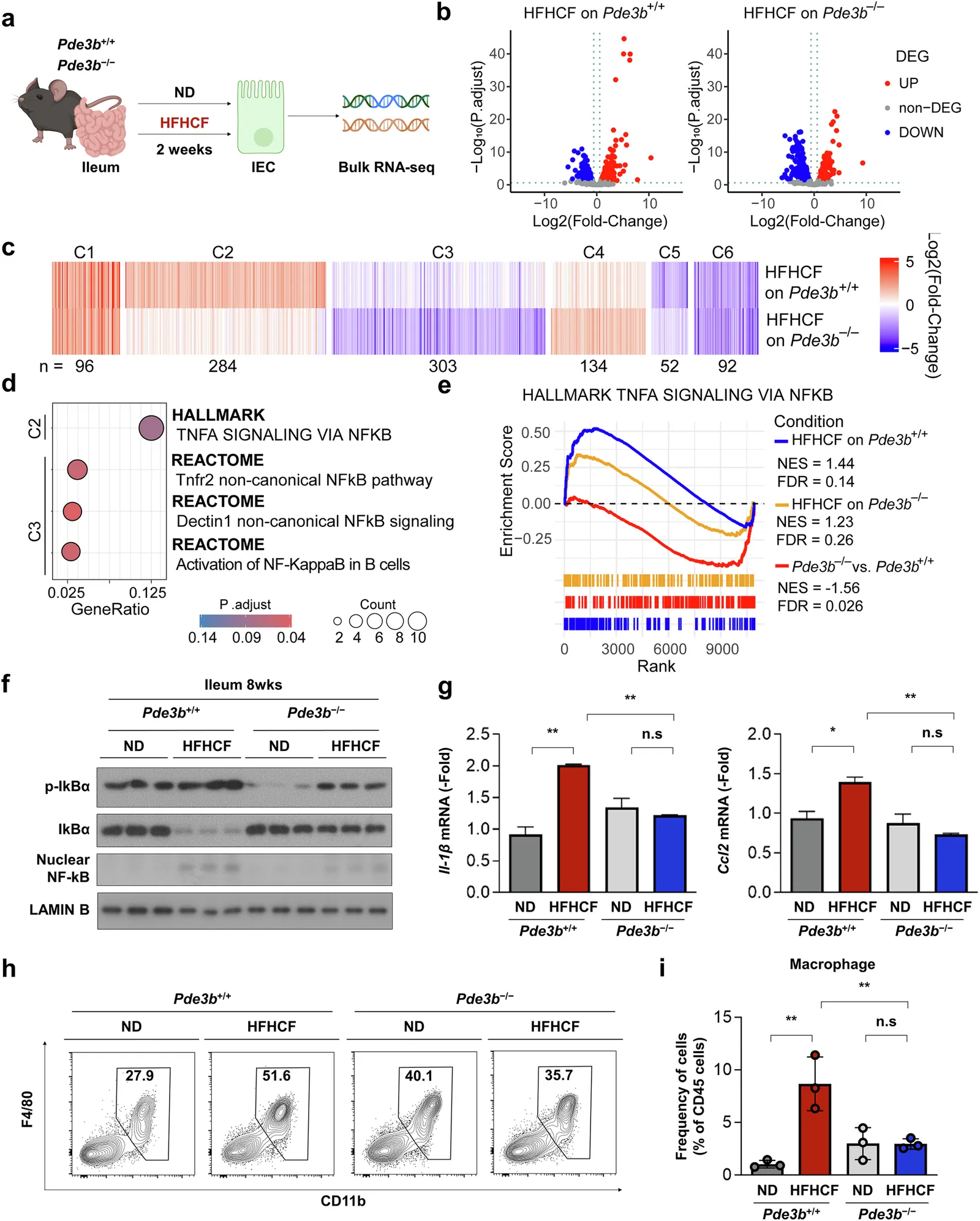
PDE3B drives NF-κB-mediated pro-inflammatory signaling in intestinal epithelial cells during MASH pathogenesis. **a**–**e** *Pde3b*^+/+^ and *Pde3b*^−/−^ mice were fed a normal diet (ND) or HFHCF diet for 2 weeks, followed by isolation of intestinal epithelial cells (IECs) (*n* = 3 per group). **a** Bulk RNA-sequencing (RNA-seq) scheme of IECs from *Pde3b*^+/+^ and *Pde3b*^−/−^ mice. **b** Volcano plots representing differentially expressed genes (DEGs) affected by HFHCF feeding in *Pde3b*^+/+^ (left) and *Pde3b*^−/−^ mice (right). **c** Heatmap of DEGs categorized into six distinct expression clusters (C1–C6). **d** Gene set over-representation analysis of C2 and C3, highlighting the significant enrichment of NF-κB signaling-related pathways. **e** Gene set enrichment analysis for the HALLMARK tumor necrosis factor-alpha (TNFα)-nuclear factor (NF)-κB signaling pathway. **f**–**i** *Pde3b*^+/+^ and *Pde3b*^−/−^ mice were fed an ND or a HFHCF diet for 8 weeks (*n* ≥ 5 per group). **f** Western blot analysis of ileum tissues with antibodies against p-IκBα, IκBα, and nuclear NF-κB, LAMIN B as loading control of nuclear extracts. **g** Relative *Il1b* and *Ccl2* mRNA levels in ileum tissue. **h**, **i** Representative flow cytometry plots and frequencies of F4/80^+^ macrophages among CD45^+^ cells in the ileum. Data are presented as mean ± SEM. *P < 0.0332; **P < 0.0021. n.s=non-significant. FDR, false discovery rate; MASH, metabolic dysfunction-associated steatohepatitis; NES, normalized enrichment score; PDE3B, phosphodiesterase 3B.

To elucidate the molecular mechanisms underlying the improved intestinal barrier dysfunction observed in *Pde3b*^−/−^ mice, bulk RNA sequencing was performed on intestinal epithelial cells (IECs) isolated from mice fed a HFHCF diet for 2 weeks. This early time point was selected to capture the initial gene expression changes associated with alterations in gut permeability during MASH progression. Differential expression analysis revealed distinct gene sets affected by HFHCF in both *Pde3b*^+/+^ and *Pde3b*^−/−^ mice, including subsets that were either shared or unique to each genotype (Fig. 5a, b and Supplementary Fig. 7a).

On the basis of their expression patterns in response to the HFHCF diet, differentially expressed genes were grouped into six clusters (C1–C6) (Fig. 5c). Genes in clusters C1 and C6 were commonly upregulated or downregulated, respectively, in both genotypes. By contrast, clusters C2 and C5 comprised genes specifically altered in *Pde3b*^+/+^ mice, whereas clusters C3 and C4 included genes uniquely regulated in *Pde3b*^−/−^ mice. Pathway enrichment analysis revealed that clusters C2 and C3 were strongly associated with NF-κB signaling-related processes (Fig. 5d and Supplementary Fig. 7b). Notably, *Pde3b*^−/−^ mice exhibited downregulation of multiple key inflammatory mediators within these clusters (highlighted in bold; Supplementary Fig. 7c). Furthermore, gene set enrichment analysis for tumor necrosis factor-alpha (TNF-α) signaling via NF-κB showed significantly reduced activation in *Pde3b*^−/−^ mice compared with *Pde3b*^+/+^ controls (Fig. 5e).

At the molecular level, p-IκBα/IκBα ratios and nuclear NF-κB levels were markedly elevated in the ileum of *Pde3b*^+/+^ MASH-model mice, consistent with enhanced NF-κB signaling activity. These increases were significantly attenuated in *Pde3b*^−/−^ mice, suggesting that PDE3B deficiency suppresses intestinal NF-κB pathway activation (Fig. 5f and Supplementary Fig. 7d,e). In parallel, *Pde3b*^−/−^ mice showed significantly reduced expression of pro-inflammatory mediators, including *Il1b* and *Ccl2*, as well as lower infiltration of lamina propria macrophages compared with *Pde3b*^+/+^ mice (Fig. 5g–i).

Collectively, these findings support a role for PDE3B in sustaining NF-κB-associated inflammatory responses in IECs during MASH progression. By modulating this pathway, PDE3B contributes to intestinal barrier function and inflammation in the context of MASH.

### Pharmacological PDE3 inhibition preserves intestinal barrier integrity in a PDE3B-dependent manner during MASH

**Fig. 6:**
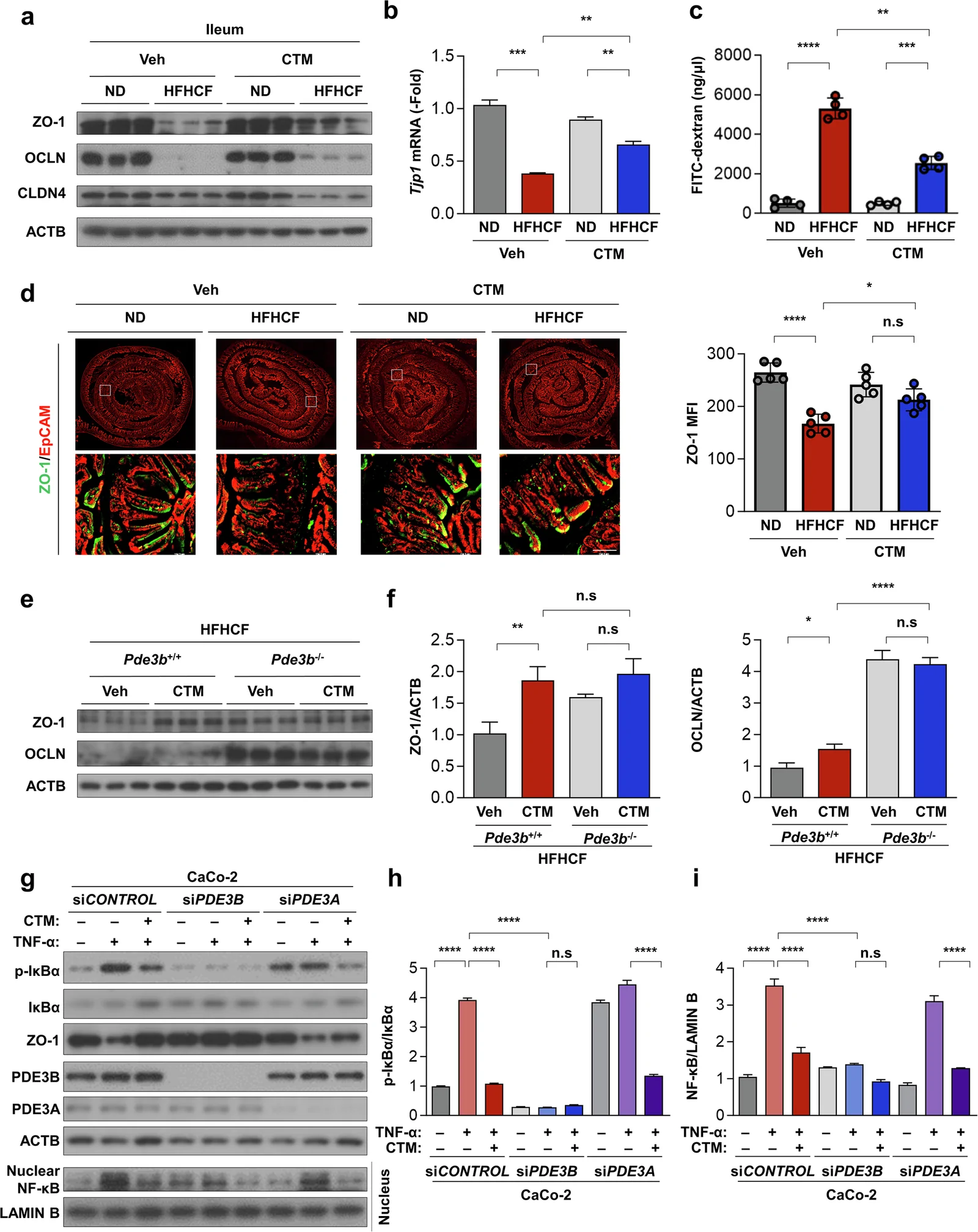
Pharmacological PDE3 inhibition by cilostamide enhances intestinal tight junction integrity and reduces permeability. **a**–**d** C57BL/6J mice were fed a normal diet (ND) or HFHCF diet for 4 weeks, followed by treatment with either vehicle (Veh) or cilostamide (CTM; 3 mg/kg) for an additional 4 weeks while continuing the respective diets. **a** Western blot analysis of ileum tissues with antibodies against ZO-1, OCLN, CLDN4, and ACTB as a loading control (*n* = 5 per group). **b** Relative *Tjp1* mRNA levels in ileum tissue. **c** Plasma fluorescein isothiocyanate (FITC)-dextran fluorescence as a measure of intestinal permeability (*n* = 4 per group). **d** Immunofluorescence staining of ZO-1 and epithelial cell adhesion molecule (EpCAM) in ileum tissues, with quantification of Zo-1-positive areas using ImageJ. **e** Representative immunoblots of the tight junction proteins ZO-1 and occludin in ileal tissue lysates from *Pde3b*^+/+^ and *Pde3b*^−/−^ mice subjected to a 4-week HFHCF diet feeding followed by 4 weeks of continued diet with vehicle or cilostamide treatment. **f** Densitometric quantification of ZO-1 and occludin normalized to the indicated loading control. **g**–**i** Caco-2 cells transfected with si*PDE3B* or si*PDE3A* were treated with tumor necrosis factor-alpha (TNF-α), with or without CTM (1 μM), for 48 h. Immunoblot analyses were performed on Caco-2 cell lysates (and nuclear fractions where indicated), followed by densitometric quantification. Data are presented as mean ± SEM. *P < 0.0332; **P < 0.0021; ***P < 0.0002; and ****P < 0.0001. n.s=non-significant. ACTB, β-actin; BAT, brown adipose tissue; MASH, metabolic dysfunction-associated steatohepatitis; MFI, mean fluorescence intensity; OCLN, occludin; PDE3B, phosphodiesterase 3B; WAT, white adipose tissue; ZO-1, zonula occludens-1.

Building on the observation that PDE3B deficiency enhances intestinal barrier function by preserving tight junction integrity and suppressing NF-κB-mediated inflammation, we next explored whether cilostamide could similarly improve intestinal permeability in MASH-model mice. Western blot analysis of ileal tissue revealed increased expression of the tight junction proteins ZO-1 and occludin in cilostamide-treated mice compared with vehicle-treated MASH controls (Fig. 6a, b and Supplementary Fig. 8a). In parallel, FITC-dextran permeability assays showed a marked decrease in intestinal leakage following cilostamide treatment, indicating improved barrier function (Fig. 6c). Immunofluorescence staining further supported these findings, showing enhanced ZO-1 localization in the intestinal epithelium of cilostamide-treated mice (Fig. 6d).

To complement the in vivo findings and to assess epithelial-intrinsic effects under metabolic inflammatory stress, we used a Caco-2 monolayer model. TNF-α stimulation was used to induce NF-κB activation, which is known to disrupt tight junctions and impair epithelial barrier integrity^28^. Cilostamide significantly increased the expression of tight junction proteins under TNF-α stimulation (Supplementary Fig. 8b). Consistently, cilostamide-treated monolayers exhibited elevated transepithelial electrical resistance, indicating improved barrier function (Supplementary Fig. 8c). FITC-dextran flux assays further confirmed reduced paracellular permeability in cilostamide-treated cells compared with untreated controls (Supplementary Fig. 8d). Together, these data support that PDE3 inhibition can preserve epithelial barrier integrity in a Caco-2 monolayer model under inflammatory conditions.

To independently validate these pharmacological observations, we performed parallel experiments in PDE3B-deficient Caco-2 cells. Under TNF-α challenge, PDE3B-deficient cells exhibited markedly reduced p-IκBα/IκBα ratios, diminished nuclear translocation of NF-κB, and significantly lower mRNA expression of pro-inflammatory cytokines such as *IL1B* and *TNF*, compared with controls (Supplementary Fig. 8e,f). Functionally, the TNF-α-induced increase in FITC-dextran permeability observed in wild-type cells was significantly attenuated in PDE3B-deficient monolayers (Supplementary Fig. 8g), and TNF-α-mediated reductions in transepithelial electrical resistance were prevented (Supplementary Fig. 8h). Consistent with these functional improvements, ZO-1 and occludin protein levels remained higher in PDE3B-deficient cells following TNF-α exposure, indicating preserved tight junction integrity (Supplementary Fig. 8i).

To further determine whether the in vivo barrier-improving effect of cilostamide depends on PDE3B, we assessed ileal tight junction proteins under HFHCF feeding with or without cilostamide in *Pde3b*^+/+^ and *Pde3b*^−/−^ mice. In HFHCF-fed wild-type mice, cilostamide increased ZO-1 and occludin compared with vehicle controls, whereas *Pde3b*^−/−^ mice showed relatively preserved tight junction protein expression with no additional induction by cilostamide (Fig. 6e, f). This non-additive pattern is consistent with cilostamide acting, at least in part, through a PDE3B-dependent pathway to support intestinal barrier integrity in vivo.

Given tissue-dependent differences in PDE3 isoform expression, we next profiled PDE3A and PDE3B across relevant tissues. PDE3B predominated in the liver with minimal PDE3A expression (Supplementary Fig. 2b), whereas both isoforms were comparably expressed in the ileum (Supplementary Fig. 8j). To determine whether the effect of cilostamide in epithelial cells could be attributed to PDE3A inhibition rather than PDE3B, we performed isoform-specific knockdown experiments in Caco-2 cells. In control cells, TNF-α robustly increased p-IκBα and nuclear NF-κB while reducing ZO-1 expression; however, these responses were markedly blunted upon PDE3B depletion (Fig. 6g–i). Under PDE3B knockdown, cilostamide failed to further suppress NF-κB signaling or restore ZO-1, indicating that its epithelial anti-inflammatory and barrier-protective effects require PDE3B expression. By contrast, PDE3A knockdown did not attenuate TNF-α-induced NF-κB activation nor preserve ZO-1 expression. Importantly, cilostamide retained its ability to suppress NF-κB signaling and enhance ZO-1 in PDE3A-depleted cells. Therefore, in this epithelial monolayer model, cilostamide’s epithelial anti-inflammatory actions are functionally dependent on inhibition of PDE3B rather than PDE3A.

Collectively, these findings demonstrate that pharmacological PDE3 inhibition with cilostamide preserves intestinal barrier integrity and reduces gut permeability during MASH progression in a PDE3B-dependent manner.

### PDE3B inhibition attenuates NF-κB activation via PKA signaling under MASH conditions

**Fig. 7:**
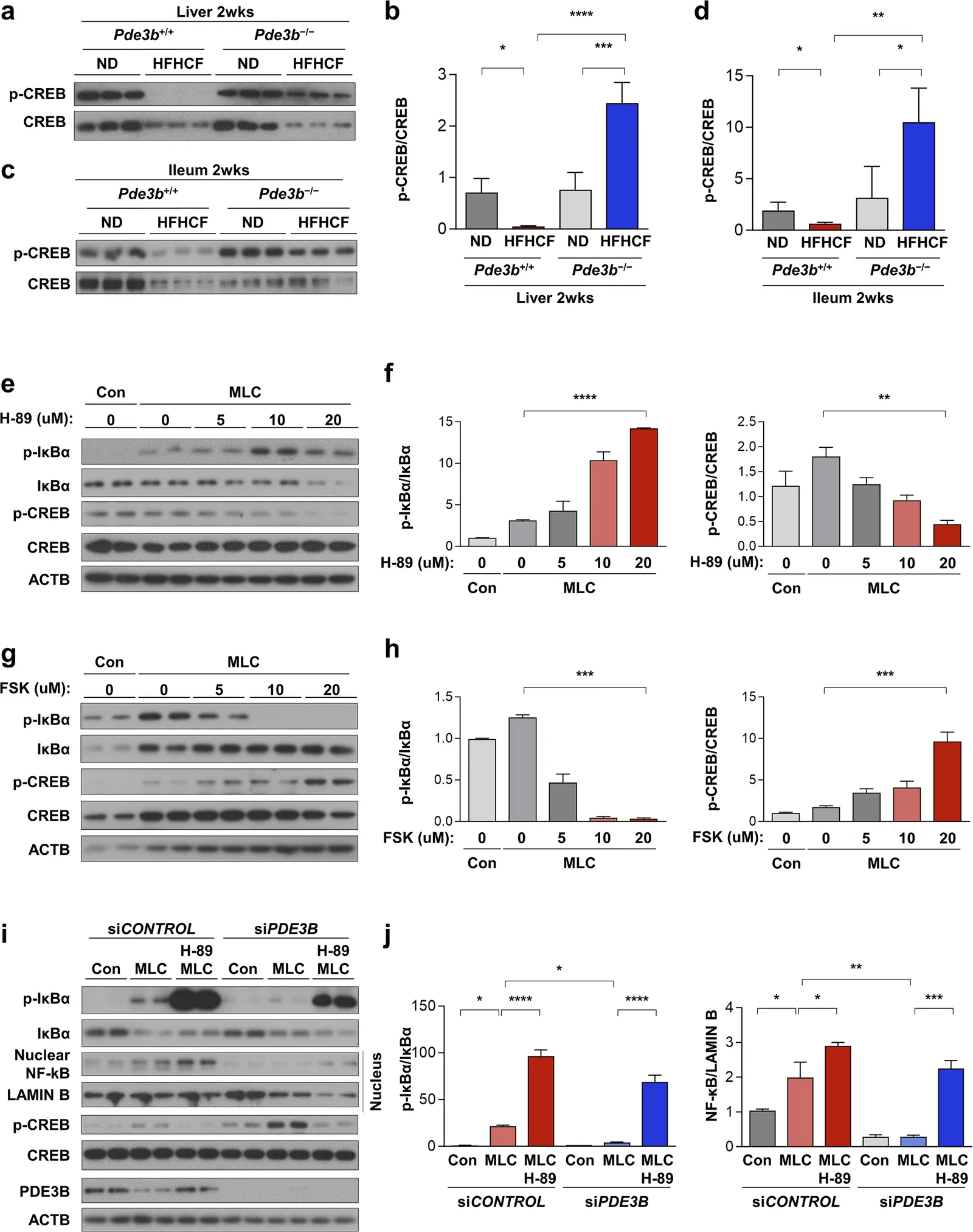
PDE3B modulates NF-κB activation through PKA signaling. **a**–**d** *Pde3b*^+/+^ and *Pde3b*^−/−^ mice in 2-weeks HFHCF model (cohort no. 12). **a**, **b** Immunoblot analysis of indicated proteins in liver tissues, with densitometric quantification. **c**, **d** Immunoblot analysis of indicated proteins in ileum tissues, with densitometric quantification. **e**–**h** Caco-2 cells were treated with metabolic dysfunction-associated steatohepatitis-like cocktail (MLC), with or without H-89 or forskolin (FSK) for 24 h. **e**, **g** Western blot analysis of cells with antibodies against indicated proteins. **f**, **h** Densitometric quantification of immunoblot in parts **e** and **g**, respectively. **i**, **j** Caco-2 cells transfected with si*PDE3B* were treated with MLC, with or without H-89, for 24 h. **i**, **j** Immunoblot analysis of indicated proteins, with densitometric quantification. Data are presented as mean ± SEM. *P < 0.0332; **P < 0.0021; ***P < 0.0002; and ****P < 0.0001. Con, control; ND, normal diet; NF-κB, nuclear factor-κB; PDE3B, phosphodiesterase 3B; PKA, protein kinase A.

Building on our findings that PDE3B loss or cilostamide treatment preserves intestinal barrier integrity and attenuates NF-κB-associated inflammatory readouts, we next sought to define the signaling mechanisms linking PDE3B with NF-κB regulation under metabolic inflammatory conditions. To test whether the cAMP–PKA pathway is engaged in vivo in association with PDE3B deficiency during MASH, we examined phosphorylation of CREB, a canonical downstream readout of PKA activity, in liver and ileal tissues. In the liver, PDE3B-deficient mice exhibited higher phosphorylation levels of CREB compared with wild-type controls under MASH conditions (Fig. 7a, b). Similarly, ileal tissues from *Pde3b*^−/−^ mice showed elevated phosphorylation of CREB relative to wild-type counterparts (Fig. 7c, d). To functionally interrogate whether PKA activity modulates NF-κB regulation in epithelial cells under metabolic inflammatory conditions, we used an MLC model in Caco-2 monolayers and performed pharmacological perturbation of PKA. Inhibition of PKA with H-89 under MLC conditions resulted in a dose-dependent increase in p-IκBα levels, accompanied by decreased phosphorylation of CREB (Fig. 7e, f). Conversely, activation of the cAMP–PKA pathway with forskolin (FSK) led to a dose-dependent reduction in p-IκBα and an increase in p-CREB levels (Fig. 7g, h). Together, these perturbation experiments identify PKA activity as a key modulatory node for NF-κB-associated inflammatory signaling in IECs.

We next examined how PDE3B interfaces with PKA signaling in this setting. PDE3B knockdown increased phosphorylation of PKA substrates relative to control cells, consistent with enhanced cAMP–PKA signaling upon loss of PDE3B-mediated cAMP hydrolysis^31,32^ (Supplementary Fig. 8k). Notably, PKA inhibitor H-89 abolished CREB phosphorylation in both control and PDE3B-deficient cells, confirming effective PKA blockade in this system. Importantly, PKA inhibition increased p-IκBα levels and nuclear translocation of NF-κB in both control and PDE3B-depleted cells, indicating that PKA blockade overrides the NF-κB-restraining phenotype conferred by PDE3B depletion (Fig. 7i, j). These pharmacological epistasis experiments support that PKA activity is required for the NF-κB-restraining phenotype associated with PDE3B loss.

Together, these in vitro and in vivo findings support a model in which PDE3B limits cAMP–PKA signaling, whereas loss of PDE3B enhances PKA activity to attenuate NF-κB activation during MASH pathogenesis.

## Discussion

In this study, we provide convergent genetic and pharmacological evidence supporting PDE3B as a therapeutically tractable multi-organ node that links intestinal barrier integrity with hepatic inflammatory-fibrotic features in diet-induced MASH models. Building on previous studies implicating PDE3B in hepatic lipid and glucose metabolism, adipose-derived regulation of liver metabolism, and systemic metabolic control, our findings extend the PDE3B–MASH literature in three respects. First, although PDE3B has been characterized in hepatocytes as a regulator of glucose, triglyceride, and cholesterol metabolism^33^ and as a downstream effector of adipose-derived^34,35^ and hypothalamus-derived^36^ metabolic inputs, its role in the intestinal compartment has not been functionally interrogated under MASH conditions. Second, although NF-κB has been reported to transcriptionally repress *Pde3b* in liver through NF-κB-binding sites identified in the *Pde3b* promoter^37^, we identify post-transcriptional loss of PDE3B protein under chronic metabolic stress without concordant transcript-level suppression, revealing an additional regulatory layer distinct from this transcriptional axis. Third, although previous pharmacological studies in fatty liver disease have largely relied on non-selective multi-PDE inhibition with limited mechanistic specificity^38^, our study combines PDE3B-targeted genetic loss-of-function, pharmacological PDE3 inhibition, and isoform-discriminating in vitro epistasis experiments to distinguish PDE3B-linked effects from potential PDE3A contribution. Together, these findings extend the PDE3B field beyond a hepatocyte-centered and adipose-centered framework and position intestinal epithelium as a previously underappreciated compartment in PDE3B-associated gut–liver axis regulation during MASH.

A notable finding of our study was the pronounced discordance between PDE3B protein and mRNA expression in MASH. PDE3B protein was selectively diminished in liver biopsies from patients and across multiple diet-induced mouse models, despite preserved or even elevated transcript levels (Fig. 1 and Supplementary Fig. 1a–i), suggesting post-transcriptional regulation. This pattern was recapitulated in Caco-2 monolayers exposed to MLC conditions, in which PDE3B protein abundance decreased without a corresponding decrease in *PDE3B* mRNA expression, indicating post-transcriptional control. Notably, pharmacological inhibition of autophagy partially restored PDE3B protein levels, whereas proteasome blockade had minimal impact (Supplementary Fig. 1j–o), supporting a contribution of lysosome-associated protein turnover under metabolic and inflammatory stress. Although the precise lysosomal route remains to be defined, this interpretation is consistent with recent evidence linking the PDE3B–cAMP axis to autophagy-associated liver injury in an independent translational setting^39^. Together, these findings identify post-transcriptional PDE3B protein loss as an additional regulatory layer during MASH progression, distinct from previously described NF-κB-mediated transcriptional repression of *Pde3b*. Importantly, the protective effects observed after genetic PDE3B deletion or cilostamide-mediated PDE3 inhibition suggest that MASH-associated PDE3B downregulation represents a partial and dynamically regulated loss of PDE3B control, rather than complete pathway extinction, thereby leaving a therapeutically tractable signaling node.

Mechanistically, our data support PDE3B as an important modulatory node that shapes NF-κB-associated inflammatory output in both the intestinal epithelium and the liver under HFHCF-driven metabolic stress^40,41^. PDE3B deficiency or inhibition attenuated the loss of tight junction proteins (ZO-1 and occludin), enhanced transepithelial barrier function, and markedly reduced portal endotoxin exposure, consistent with diminished gut-derived inflammatory input to the liver (Figs. 2–6). Notably, ileal PDE3B protein reduction emerged early during HFHCF feeding, before overt hepatic pathology and before detectable hepatic PDE3B loss, supporting early intestinal involvement in diet-induced MASH progression. In the liver, PDE3B expression was predominantly localized to hepatocytes, with minimal expression in Kupffer cells, monocyte-derived macrophages, and neutrophils (Supplementary Fig. 3b,c). Together with reduced hepatic NF-κB signaling, macrophage recruitment, Kupffer cell repopulation dynamics, and fibrotic activation in the *Pde3b*^−/−^ model, these findings suggest that PDE3B-associated hepatic inflammation is unlikely to be driven primarily by immune-cell-intrinsic PDE3B loss, but may instead involve non-immune hepatic compartments, gut-derived inputs, or both. However, the global nature of the *Pde3b*^−/−^ model limits definitive assignment of tissue-specific causality. Collectively, these findings position PDE3B as a therapeutically tractable multi-organ node whose loss-of-function or pharmacological inhibition coordinately attenuates intestinal barrier dysfunction, gut-derived inflammatory input, and hepatic inflammatory-fibrotic remodeling during diet-induced MASH.

At the signaling level, our data identify PKA as a functional downstream effector linking PDE3B activity to NF-κB regulation under MASH-associated metabolic inflammatory stress. Bidirectional pharmacological modulation in Caco-2 cells showed that PKA inhibition enhanced p-IκBα and nuclear NF-κB accumulation, whereas forskolin-mediated PKA activation suppressed NF-κB signaling (Fig. 7e–h). Moreover, PDE3B depletion increased phosphorylation of PKA substrates, and PKA blockade overrode the NF-κB-restraining phenotype conferred by PDE3B loss (Fig. 7i, j). Together with increased CREB phosphorylation in the liver and ileum of PDE3B-deficient mice (Fig. 7a–d), these findings support a model in which PDE3B limits cAMP–PKA signaling, whereas PDE3B loss or inhibition engages a PKA-dependent pathway that constrains NF-κB activation under MASH-associated inflammatory conditions.

Therapeutically, cilostamide effectively recapitulated key protective features of PDE3B deficiency, including reduced hepatic inflammation and fibrosis and improved intestinal barrier integrity (Figs. 3 and 6). Because cilostamide inhibits both PDE3 isoforms, we directly tested whether its epithelial effects were linked to PDE3B rather than PDE3A. Our in vivo genetic epistasis studies showed that cilostamide increased ileal ZO-1 and occludin in wild-type mice, whereas these proteins were already preserved in *Pde3b*^−/−^ mice and were not further induced by cilostamide, a non-additive pattern consistent with a PDE3B-linked mechanism (Fig. 6e, f). In Caco-2 cells, PDE3B depletion, but not PDE3A depletion, attenuated TNF-α-induced NF-κB activation and preserved ZO-1 expression; moreover, cilostamide failed to further enhance these effects after PDE3B depletion but remained effective in PDE3A-depleted cells (Fig. 6g–i). Together, these findings demonstrate that although cilostamide targets both PDE3 isoforms, its epithelial anti-inflammatory and barrier-protective effects are functionally mediated by PDE3B. These data mitigate isoform-specificity concerns in the epithelial context and support PDE3B as the functionally relevant mediator of cilostamide’s barrier-protective effects in vitro. Previous clinical evaluations of non-selective phosphodiesterase inhibitors (for example, pentoxifylline) or selective PDE4 or PDE2 inhibitors yielded inconsistent outcomes and undesirable side effects^42–45^. Furthermore, recent reports on cilostazol, another PDE3 inhibitor, demonstrated improvements in gut barrier or hepatic lipid metabolism but lacked direct mechanistic linkage to PDE3B^46,47^. Our study uniquely provides convergent evidence implicating PDE3B-driven NF-κB activation across both hepatic and intestinal tissues, offering a clear mechanistic rationale for PDE3B-selective inhibition strategies.

Despite these promising findings, several limitations should be noted. First, because the present study relies largely on global *Pde3b* loss-of-function and pharmacological PDE3 inhibition, definitive tissue-specific causality and directional relationships within the gut–liver axis cannot be established. Future studies incorporating intestine-specific PDE3B manipulation, barrier-targeted rescue, and microbiota transfer approaches will be required to define whether intestinal PDE3B dysfunction is causal, permissive, or amplifying in MASH progression. Second, although our perturbation and epistasis data support the PDE3B–cAMP-PKA axis that restrains NF-κB activation, we did not directly test whether NF-κB attenuation is necessary or sufficient for the observed barrier-protective phenotype. Third, the upstream mechanisms driving the earlier reduction of ileal PDE3B relative to hepatic PDE3B remain incompletely defined and may involve early diet-induced alterations in gut microbiota and microbial metabolites. Fourth, although hepatocyte-enriched PDE3B expression and minimal PDE3B expression in hepatic immune subsets suggest a non-immune-cell-intrinsic mechanism, direct hepatocyte–immune-cell co-culture or related interaction studies will be required to establish causality. Finally, some translational datasets, including portal vein LPS and patient protein analyses, were limited by sample availability; accordingly, these findings should be regarded as exploratory and confirmed in larger, well-powered, independently recruited cohorts. In addition, although the cilostamide regimen used here was supported by prior literature and by on-target pathway engagement in our system^18,19,48,49^, further dose optimization, pharmacokinetic and pharmacodynamic characterization, and long-term safety evaluation will be necessary before clinical translation.

In conclusion, our data support PDE3B as a post-transcriptionally regulated multi-organ node that shapes inflammatory signaling and barrier-associated dysfunction across the gut–liver axis in diet-induced MASH. By linking PDE3B activity to a cAMP-PKA-dependent restraint of NF-κB output in IECs and demonstrating convergent genetic and pharmacological protection in vivo, this study nominates PDE3-targeted, ideally PDE3B-selective, inhibition as a therapeutically tractable strategy for preclinical MASH, while highlighting the need for tissue-specific causal testing and expanded translational validation.

## Supplementary information

Supplementary information
