# Supplementary material for: PDE3B as a multi-organ therapeutic node in the gut–liver axis of metabolic dysfunction-associated steatohepatitis
Source: Exp Mol Med. 2026 Sep 1;58(9):2873–87. doi: 10.1038/s12276-026-01811-2 (PMC13633239; doi:10.1038/s12276-026-01811-2)
Supplement: Supplementary file 1 [file 12276_2026_1811_MOESM1_ESM.pdf]

PDE3B as a Multi-Organ Therapeutic Node in the Gut–Liver Axis of Metabolic Dysfunction-associated Steatohepatitis

Yu Seol Lee, Chang-Yun Jeong, Youn Wook Chung, Yoseob Lee, Da Hyun Lee, Ji Yun Bang, Da Ye Kim, Haram Lee, Soohwan Oh, Jinhyuk Bhin, Ji-Hwan Ryu, June-Yong Lee, Soo Han Bae

Table of contents

Supplementary materials and methods........ **2**

Supplementary Fig 1........ **10**

Supplementary Fig 2........ **11**

Supplementary Fig 3........ **12**

Supplementary Fig 4........ **13**

Supplementary Fig 5........ **14**

Supplementary Fig 6........ **15**

Supplementary Fig 7........ **16**

Supplementary Fig 8........ **17**

Supplementary Table 1........ **18**

Supplementary Table 2........ **20**

Supplementary Table 3........ **22**

Supplementary Table 4........ **24**

Supplementary Table 5........ **25**

Supplementary Table 6........ **26**

Supplementary Table 7........ **27**

Reference........ **28**

Supplementary materials and methods

MASH Mouse Models

Following a 1-week acclimation period, mice were subjected to three distinct MASH-inducing diets:

1) High-fat, high-cholesterol diet (HFHCD; DooYeol Biotech, 600024) containing 27.5% palm oil, 2.5% cholesterol, and 2% cholic acid, combined with 2.3% fructose water for 2 or 8 weeks.
2) Choline-deficient, L-amino acid-defined, high-fat diet (CDAHFD; Research Diets, A06071302) for 12 weeks.

3) Methionine-choline-deficient diet (MCD; Research Diets, A02082002BR) for 4 weeks.

The Model 1 was primarily used in our study due to its ability to induce both significant hepatic steatosis and intestinal barrier dysfunction, closely mimicking the gut-liver axis disruption observed in human MASH.[1]

Animal cohorts

Seven independent 8-week PDE3B WT/KO cohorts (Sets 1–7; per-arm n = 3–7, totaling 31–32 per-arm), four 2-week cohorts (Sets 1–4; per-arm n = 3–6, totaling 16–20 per-arm), and one 4-week cohort (Set 1; per-arm n = 5) were generated. Three independent pharmacology cohorts (8 weeks; male C57BL/6J mice on HFHCF ± cilostamide; per-arm n = 14) were also included. The detailed allocation of each cohort to specific assays is provided Supplementary Tables 7, and the cohort labels used in the experiments are included in the corresponding figure legends. Details of mice used are provided in the Supplementary Table 4.

Pharmacological Intervention

To evaluate the effects of pharmacological PDE3 inhibition, cilostamide (3 mg/kg) or vehicle was administered via oral gavage starting at week 4 of the HFHCF regimen. The cilostamide dosing regimen (3 mg/kg, oral gavage) was selected based on prior *in vivo* reports employing cilostamide for PDE3 inhibition in mice within a tolerated dosing range[2,3]. At the end of the experimental period, mice were euthanized, and blood serum, epididymal fat, liver, and intestinal tissues were collected for analysis.

Human Liver Samples

Human liver tissues were obtained from patients at Severance Hospital under the approval of the Institutional Review Board (IRB no. 4-2019-0713). Liver biopsies were performed on three patients with or without MASH, diagnosed based on histological evaluation. All procedures for human sample collection and analysis were conducted in compliance with the ethical guidelines of Yonsei University College of Medicine and adhered to the principles outlined in the Declaration of Helsinki.

PDE3B Expression Analysis Using Gene Expression Omnibus (GEO) database

PDE3B expression levels were analyzed using publicly available microarray data from the GEO database under accession number GSE48452 and GSE192740. This dataset contains transcriptomic profiles relevant to liver and metabolic dysfunction. Data preprocessing, including background correction and normalization, was performed using GEO2R, an interactive tool provided by GEO. PDE3B expression was extracted and analyzed across relevant experimental groups to determine changes in gene expression under pathological conditions. Results are presented as normalized expression values. Details of software and GEO sets used are provided in the Supplementary Table 5 and 6.

Immunoblot Analysis

Cells and tissues were lysed in buffer containing 50 mM Tris-HCl (pH 7.5), 150 mM NaCl, 1% NP-40, 1 mM PMSF, 1 mM 4-(2-aminoethyl)benzenesulfonyl fluoride, 1 mg/mL aprotinin, and 1 mg/mL leupeptin. The homogenates were centrifuged at 20,000 × g for 15 minutes at 4°C to collect the supernatants. Protein concentrations were determined using the Bradford assay (Bio-Rad). Equal amounts of protein were separated on SDS-PAGE, transferred onto PVDF membranes, and blocked in 5% skim milk for 1 hour. Membranes were incubated overnight at 4°C with primary antibodies (1:2000) and subsequently with HRP-conjugated secondary antibodies (1:5000) for 1 hour at room temperature. Protein bands were detected using enhanced chemiluminescence reagents (Thermo Fisher Scientific). Detailed antibody information is provided in the Supplementary Table 1. To standardize group-level measurements and minimize between gel variance, a predefined equal-mass pooling strategy was adopted (as previously described [4]). Equal protein from tissue of mice within same group was combined to generate one biological replicate; per arm, independent pools form non-overlapping animals were analyzed. Technical duplicates or triplicates were averaged within pool; statistics were performed on biological replicates (independent pools or individual

animals, as indicated).

Isolation of Lamina Propria Cells

The ileum was carefully cleaned of mesenteric fat and Peyer's patches, washed in cold divalent cation-free HBSS (supplemented with HEPES, penicillin/streptomycin, and sodium pyruvate), and cut longitudinally. The tissue was incubated with HBSS containing 1 mM DTT at 37°C for 15 minutes with shaking (200 rpm) to remove mucus, followed by incubation with 5 mM EDTA at 37°C for 10 minutes to isolate epithelial cells. Minced tissue fragments were digested in RPMI medium containing 1 mg/mL collagenase D (Roche), 100 µg/mL DNase I (Sigma), and 0.05 U/mL dispase (Worthington) with constant stirring at 37°C for 45 minutes. The suspension was filtered and centrifuged to collect lamina propria cells, which were separated using a 30%/70% Percoll gradient. Details of the antibodies and chemicals used are provided in the Supplementary Table 1 and 2.

Isolation of Hepatic Non-Parenchymal Cells

Livers were perfused with pre-warmed HBSS for 2 minutes, followed by perfusion with 0.2 mg/mL collagenase A in HBSS for 8 minutes at 5 mL/min. The liver was excised, decapsulated, and shaken gently to release cells. The suspension was passed through a 100 µm mesh filter, centrifuged at 500 rpm for 3 minutes, and the supernatant was collected. After additional centrifugation at 1700 rpm for 5 minutes, the pellet was resuspended in RPMI medium containing 10% FBS and 1% penicillin/streptomycin. The suspension was underlaid with 5 mL of a 30% Percoll gradient and centrifuged. The cell pellet was treated with red blood cell lysis buffer and washed before the final centrifugation at 1700 rpm for 5 minutes. Details of the antibodies and chemicals used are provided in the Supplementary Table 1 and 2.

Serum Biochemistry

Serum alanine aminotransferase (ALT), total cholesterol (TCHO), and high-density lipoprotein (HDL) were measured using FUJI DRI-CHEM SLIDE kits (Fujifilm) and analyzed on a colorimetric assay kits.

Serum LPS Measurement

Portal vein blood was collected and centrifuged at 3,000 × g for 10 minutes at 4°C to obtain serum. Serum lipopolysaccharide (LPS) levels were measured using the Mouse Lipopolysaccharide (LPS) ELISA Kit (AVIVA Systems Biology) according to the

manufacturer's instructions. Serum samples were diluted as necessary to fit within the linear range of the standard curve. Absorbance was measured at 450 nm using a microplate reader, and LPS concentrations were determined based on the standard curve generated with the provided LPS standards.

Immunohistochemistry and Histology

Tissues were fixed in 10% neutral-buffered formalin solution (Sigma Aldrich) overnight at 4°C. For immunofluorescence, fixed tissues were transferred to 30% sucrose solution until saturated, embedded in OCT medium, and cryosectioned at 14 µm thickness. Sections were blocked in PBST (0.5% Triton X-100 with 10% normal goat serum) for 1 hour and incubated with primary antibodies overnight at 4°C. The sections were washed and incubated with secondary antibodies for 2 hours, followed by DAPI staining for nuclei.

For ZO-1 quantification, the intestinal epithelium was identified based on EpCAM expression, and the EpCAM-expressing villus region was defined as the region of interest (ROI). The mean fluorescence intensity (MFI) of ZO-1 within the ROI was measured using the LAS X application.

For paraffin-embedded tissues, sections were deparaffinized, rehydrated, and stained with F4/80 antibody for immunohistochemistry. Antigen retrieval was performed, and signal visualization was achieved using DAB staining. Standard stains, including H&E, Masson's trichrome, and Sirius red, were applied to assess histological changes. Images were captured with a Nikon Coolscope microscope, and ImageJ software was used for quantitative analysis.

Hydroxyproline Measurement

Liver tissues (20 mg) were hydrolyzed in 200 µL of 12 N HCl in a Teflon-sealed pressure vial at 120°C for 3 hours. Hydrolyzed samples (10 µL) were dried at 65°C in a 96-well plate and processed using a Hydroxyproline Colorimetric Assay Kit (BioVision) according to the manufacturer's instructions.

In vivo FITC-Dextran Permeability Assay

Mice were fasted for 4 hours before oral administration of FITC-dextran (600 mg/kg). Blood was collected after 4 hours, and plasma FITC-dextran levels were measured using a SpectraMax M4 spectrophotometer (excitation: 485 nm; emission: 535 nm).

Nuclear Fractionation

Tissues were homogenized in hypotonic buffer (10 mM HEPES, pH 7.9; 10 mM KCl; 1.5 mM $MgCl_2$; 0.5 mM DTT; 0.05% NP-40) and incubated on ice for 30 minutes. The lysates were centrifuged at 3000 rpm for 10 minutes to isolate nuclei. Nuclear pellets were resuspended in hypertonic buffer (5 mM HEPES, pH 8.0; 1.5 mM $MgCl_2$; 26% glycerol; 0.2 mM EDTA; 0.5 mM DTT) and centrifuged at 24,000 × g for 30 minutes, and the resulting supernatants were subjected to analysis of immunoblotting using antibodies of NF-κB and LAMIN B.

RNA Isolation and Real-Time qPCR

Total RNA was extracted using TRIzol (Molecular Research Center) and reverse-transcribed into cDNA with a cDNA synthesis kit (Takara). Quantitative PCR was conducted using SYBR Green on an ABI PRISM 7700 system (Applied Biosystems). Gene expression levels were normalized to 18S or GAPDH mRNA. Primer sequences are detailed in the Supplementary Table 3. Equal-mass pooling was used to capture a standardized group-level transcript readout and reduce batch effects (as previously described [5]). Each arm included independent pools from non-overlapping animals; technical duplicate averaged within pool.

RT-PCR

One microgram of total RNA was reverse transcribed into cDNA using a cDNA synthesis kit (Takara). PCR was performed using Takara's 50X Advantage-GC 2 Polymerase Mix (Takara) with the following cycling parameters: denaturation at 94°C for 30 s, annealing at 60°C for 30 s, and extension at 68°C for 30 s, for 35 cycles. The primer sequences are listed in the Supplementary Table 3.

Hepatic Steatosis Measurement

Liver triglyceride content was measured with a triglyceride colorimetric assay kit (Cayman Chemical). Oil Red O staining was performed on cryosections, followed by Mayer's hematoxylin counterstaining. Briefly, Cryosections (5 μm) of liver tissues were air-dried at room temperature for at least 30 minutes and fixed in 10% formalin for 5 minutes. After fixation, sections were dehydrated in 100% propylene glycol for 5 minutes and stained with Oil Red O solution in a 60°C oven for 10 minutes. Excess stain was removed with 85% propylene glycol, and sections were counterstained with Mayer's hematoxylin for 1 minute to visualize nuclei. Slides were mounted using an aqueous mounting solution and analyzed under a microscope

within 2 hours of preparation.

Isolation of RNA and library preparation for bulk RNA sequencing

Total RNA was extracted from isolated ileal intestinal epithelial cells (IECs) using TRIzol reagent. Following addition of 100% ethanol, RNA was purified using the Direct-zol RNA Microprep Kit (Zymo Research, R2061-A) according to the manufacturer's instructions. RNA quantity and integrity were assessed using an Agilent TapeStation 4150 with the High Sensitivity RNA ScreenTape Assay (Agilent, 5067-5579).

RNA-seq libraries were prepared using the NEBNext® Single Cell/Low Input RNA Library Prep Kit for Illumina (NEB, E6420S) following the manufacturer's protocol. Indexing was performed using NEBNext® Multiplex Oligos for Illumina® (Index Primers Set 1; NEB, E7335S). The final amplification cycle number was determined by qPCR using the ReverTra Ace qPCR RT Kit (TOYOBO, TOFSQ-101). Cleanup and size selection were performed using the AmpliClean™ Cleanup Kit with Magnetic Beads (NimaGen, AP-005) at each step. Final libraries were assessed using an Agilent TapeStation 4150 with the D1000 DNA ScreenTape Assay (Agilent, 5067-5582) and sequenced on an Illumina NovaSeq 6000 platform.

Bulk RNA-Seq Analysis

Raw FASTQ files were quality-checked using FastQC (v0.12.1). Adapter sequences and low-quality bases were trimmed using Cutadapt (v4.9), applying a minimum Phred quality cutoff of 20 and removing reads containing ambiguous bases. Trimmed reads were aligned to the mouse reference genome (GRCm39) using STAR (v2.7.10b).

Gene-level read counts were generated using featureCounts (v2.0.6) with GENCODE gene annotation (Ensembl vM34). Differential expression analysis was performed in R using DESeq2 (v1.44.0) with a design matrix incorporating genotype and diet. Count normalization was carried out using the DESeq2 median-of-ratios method, and P values were adjusted for multiple testing using the Benjamini–Hochberg procedure. Differentially expressed genes (DEGs) were defined as those meeting an adjusted false discovery rate (FDR) < 0.25 and an absolute log2 fold change ≥ 0.58.

For functional interpretation, over-representation analysis was performed using clusterProfiler (v4.12.6) with Hallmark and Reactome gene sets obtained from MSigDB (v7.5.1). Gene set enrichment analysis (GSEA) was conducted using fgsea (v1.30.0) based on ranked gene lists.

Heatmaps and summary plots were generated using ComplexHeatmap (v2.20.0) and ggplot2 (v3.5.1).

RNA-seq data have been deposited in the NCBI Gene Expression Omnibus (GEO) under accession number GSE285516. Details of software and GEO sets used are provided in the Supplementary Table 5 and 6.

Cell Culture and Treatment

Caco-2 cells (KCLB 30037.1) were cultured in Minimum essential medium (Eagle) (MEM; sigma) supplemented with 10% fetal bovine serum (FBS), 1% non-essential amino acids (NEAA; Gibco), and 1% penicillin/streptomycin (Biowest). Cells were maintained at 37°C in a humidified incubator with 5% CO_2. For TEER and FITC-dextran permeability assays, Caco-2 cells were seeded at a density of 3.8×10^4 cells/cm^2 on collagen-coated 24 well Transwell® inserts (polycarbonate membranes, 0.4 μm pore size; Corning). Cells were cultured for 14 days to form a monolayer, and the culture medium was replaced every 2 days. Details of cells used are provided in the Supplementary Table 4.

For experimental treatments, cells were transfected with siRNA targeting PDE3B (siPDE3B; Ambion), PDE3A (siPDE3A; 5'- GGAAGAAGAAGAAGAGAAA-3'), or scrambled control siRNA (siControl) using Lipofectamine RNAiMAX (Invitrogen) according to the manufacturer's instructions. After 48 hours of siRNA transfection, Caco-2 cells were treated with TNF-α (50 ng/mL) in the presence or absence of cilostamide (1 μM; DMSO vehicle) for 48 hours. The cilostamide concentration used for epithelial assays (1 μM; DMSO vehicle) was chosen based on commonly used cell-based conditions for pharmacological PDE3 inhibition[6,7].

To stimulate MASH-like condition, Caco-2 cells were exposed for 24 hours to the following MASH-like cues (MLC): 500 μM palmitate, 1% lipid mixture, 50 μM fructose, and 1 μg/ml LPS. Unless otherwise specified, inhibitors were added during the final 2-6 hours (100 nM bafilomycin A1; 10 μM MG132) before lysis. To manipulate cAMP-PKA signaling, cells were treated with forskolin or H-89 for 24 hours.

In vitro FITC-Dextran Permeability Assay

Following treatment, FITC-dextran (4 kDa; Sigma Aldrich) was prepared at a final concentration of 1 mg/mL in HBSS and added to the apical chamber of the Transwell system. After 2 hours of incubation at 37°C, 100 μL of the basolateral medium was collected, and

fluorescence intensity was measured using a SpectraMax M4 plate reader (Molecular Devices) with excitation at 485 nm and emission at 535 nm. The permeability of FITC-dextran was quantified as fluorescence in the basolateral chamber and expressed relative to the control.

TEER Measurement

Transepithelial electrical resistance (TEER) was measured to evaluate monolayer integrity using an EVOM2 voltohmmeter (World Precision Instruments) equipped with STX2 electrodes. TEER values were recorded immediately after completing treatments. All measurements were performed in triplicate, and background resistance (blank inserts) was subtracted. TEER values were normalized to the surface area of the Transwell insert and expressed as ohms × cm^2.

Data availability statement

The RNA sequencing data generated in this study is available under the accession number GSE285516 in the NCBI Gene Expression Omnibus (GEO).

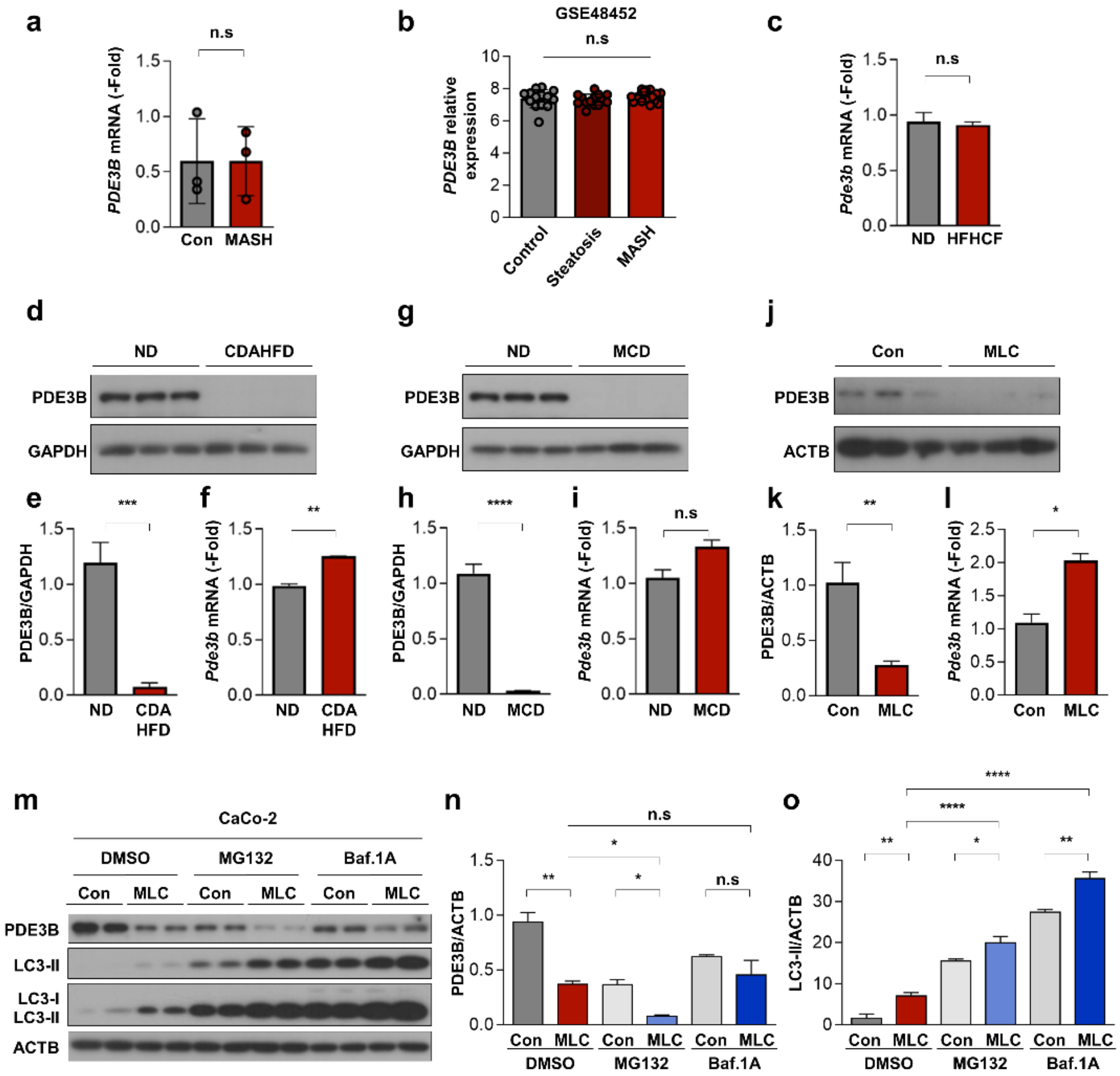

Supplementary Fig. 1. PDE3B protein expression is reduced in liver tissues of MASH patients and mouse models

(a) mRNA expression of *PDE3B* in liver biopsies from normal individuals and MASH patients (Cohort #3). (b) Analysis of *PDE3B* mRNA in human liver tissues (Control: n=14; Steatosis: n=14; MASH: n=18) based on publicly available transcriptomic data (GEO accession number GSE48452). (c) Real-time qPCR analysis of *Pde3b* mRNA in the liver of HFHCF-fed MASH mouse model (Cohort #4). (d–i) Immunoblot analysis of PDE3B in liver tissue lysates from various diet-induced MASH models, with densitometric quantification and Real-time qPCR analysis of *Pde3b* mRNA expression (Cohort #1, 2). (j–l) Immunoblot analysis of Caco-2 lysates after MASH-like cocktail (MLC), with densitometric quantification and real-time qPCR of *Pde3b* mRNA. (m) Immunoblot of PDE3B and LC3 in Caco-2 cells; ACTB served as loading control. (n, o) Densitometric quantification of PDE3B and LC3-II, respectively. Data are presented as mean ± SEM. *P < 0.0332; **P < 0.0021; ***P < 0.0002 and ****P < 0.0001. n.s=non-significant. MCD, methionine-, choline-deficient diet; CDAHFD, choline-deficient, l-amino acid-defined, high-fat diet; ND, normal diet; MLC, MASH-like cocktail.

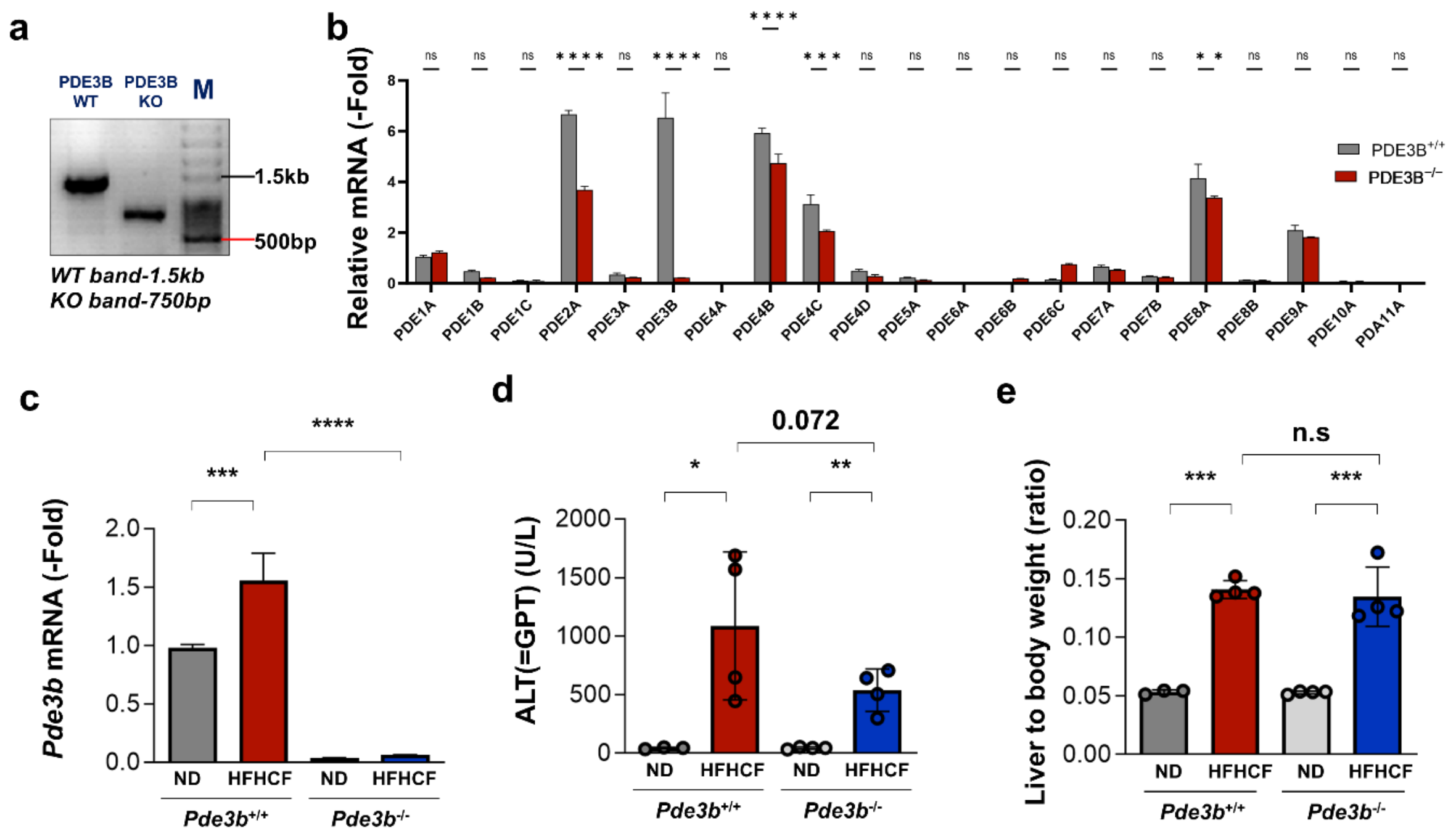

Supplementary Fig. 2. Validation of PDE3B knockout (KO) mice and phenotypic characterization in MASH development

(a) Genotyping PCR confirms wild-type (WT) and PDE3B KO genotypes. (b) qPCR analysis of PDE isoforms in liver tissue. (c) Real-time qPCR analysis of *Pde3b* mRNA of the liver in WT and KO mice during MASH induction. (d) Serum ALT. (e) Liver-to-body weight ratio. (Cohort #4) Data are presented as mean ± SEM. *P < 0.0332; **P < 0.0021; ***P < 0.0002 and ****P < 0.0001. n.s=non-significant. ND, normal diet.

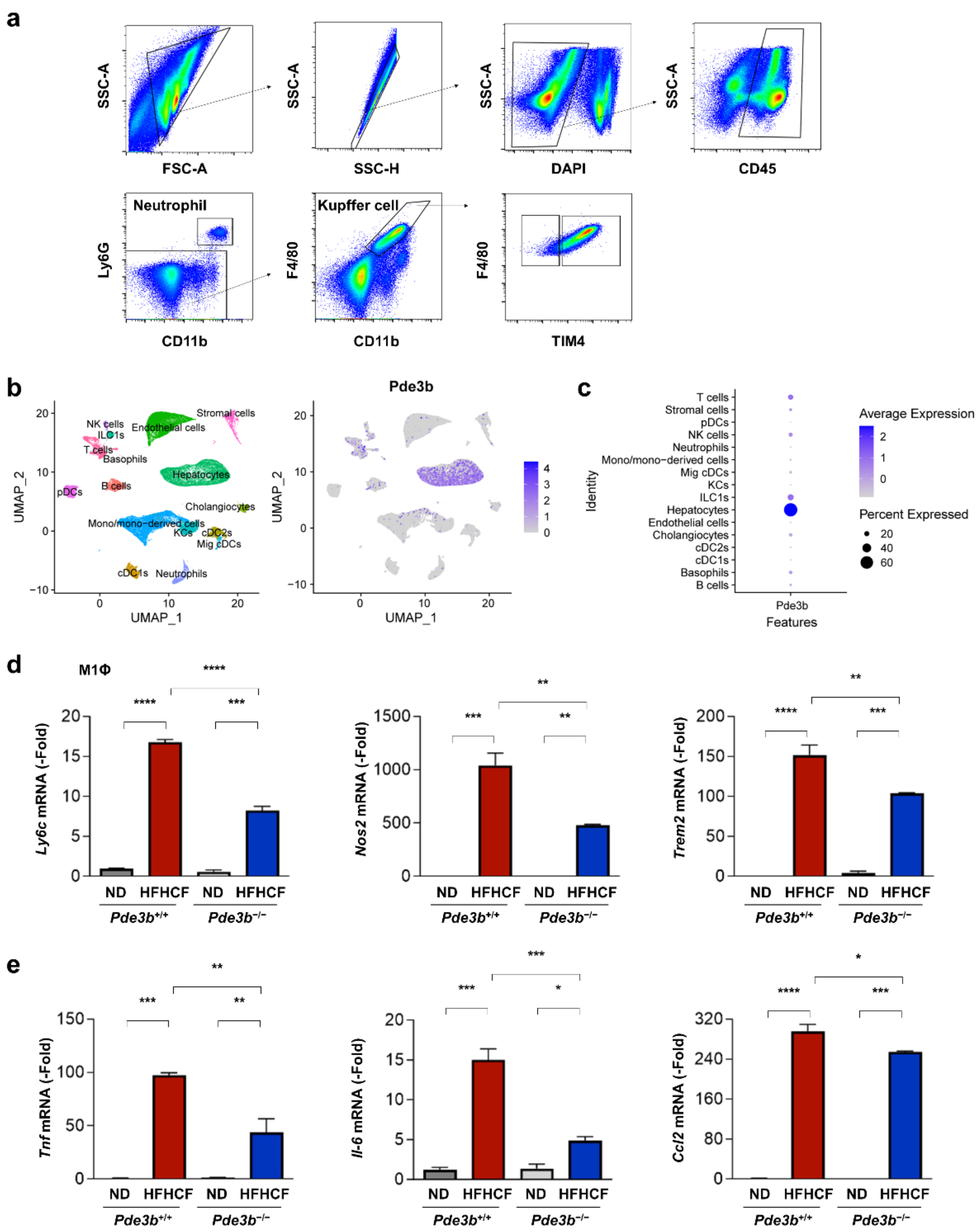

Supplementary Fig. 3. PDE3B deficiency downregulates pro-inflammatory gene expression in the liver during MASH

(a) Gating strategy for identifying Tim4^{+} resident Kupffer cells and Tim4^{-} infiltrating macrophages from CD45^{+} cells in liver tissue (related to Fig. 2C). (b, c) UMAPs showing annotations of cell types and Pde3b expression based on publicly available single cell RNA sequencing data (GEO accession number GSE192740). (d, e) Real-time qPCR analysis of indicated gene expression in liver tissues (Cohort #6). Data are presented as mean ± SEM.*P < 0.0332; **P < 0.0021; ***P < 0.0002 and ****P < 0.0001. ND, normal diet.

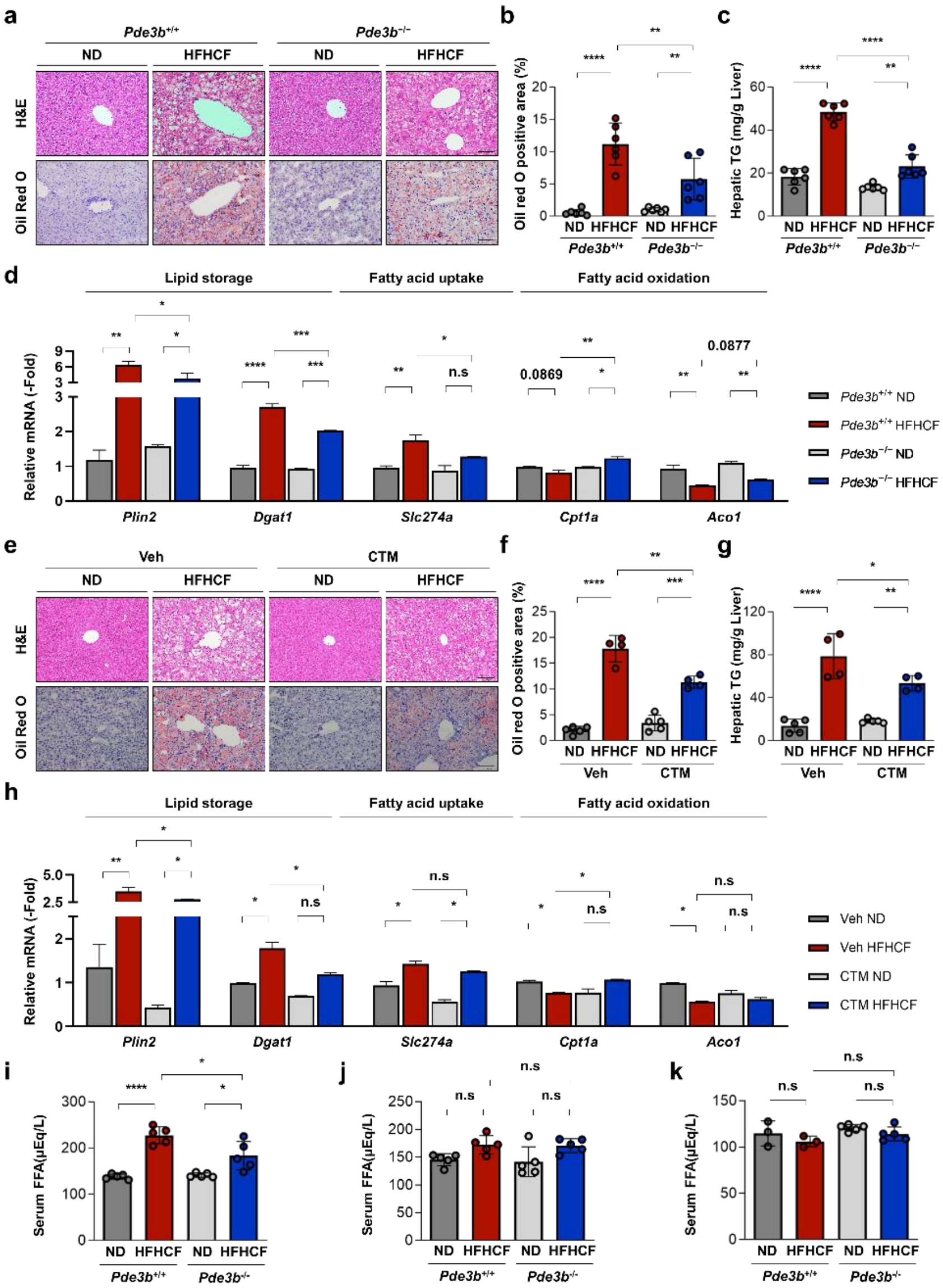

Supplementary Fig. 4. PDE3B deletion and pharmacological inhibition prevent hepatic lipid accumulation in diet-induced MASH

(a-d) *Pde3b*$^{+/+}$ and *Pde3b*$^{-/-}$ mice in 8 weeks HFHCF model (Cohort #6). (a) Representative photomicrographs of liver sections stained with H&E (top) and Oil Red O (bottom); scale bar, 100 μm. (b) Quantification of Oil Red O-stained areas. (c) Hepatic triglyceride content normalized to liver weight. (d) Real-time qPCR analysis of indicated gene expression. (e-h) C57BL/6J mice in 8 weeks HFHCF model with cilostamide (3 mg/kg) (Cohort #17). Corresponding histological, biochemical, and gene expression analyses were performed. (i-k) Serum NEFA levels in 2, 4, and 8 weeks HFHCF model, respectively (Cohort #12, 11, and 5). Data are presented as mean ± SEM. *P < 0.0332; **P < 0.0021; ***P < 0.0002 and ****P < 0.0001. n.s=non-significant. ND, normal diet. Veh, vehicle; CTM, cilostamide.

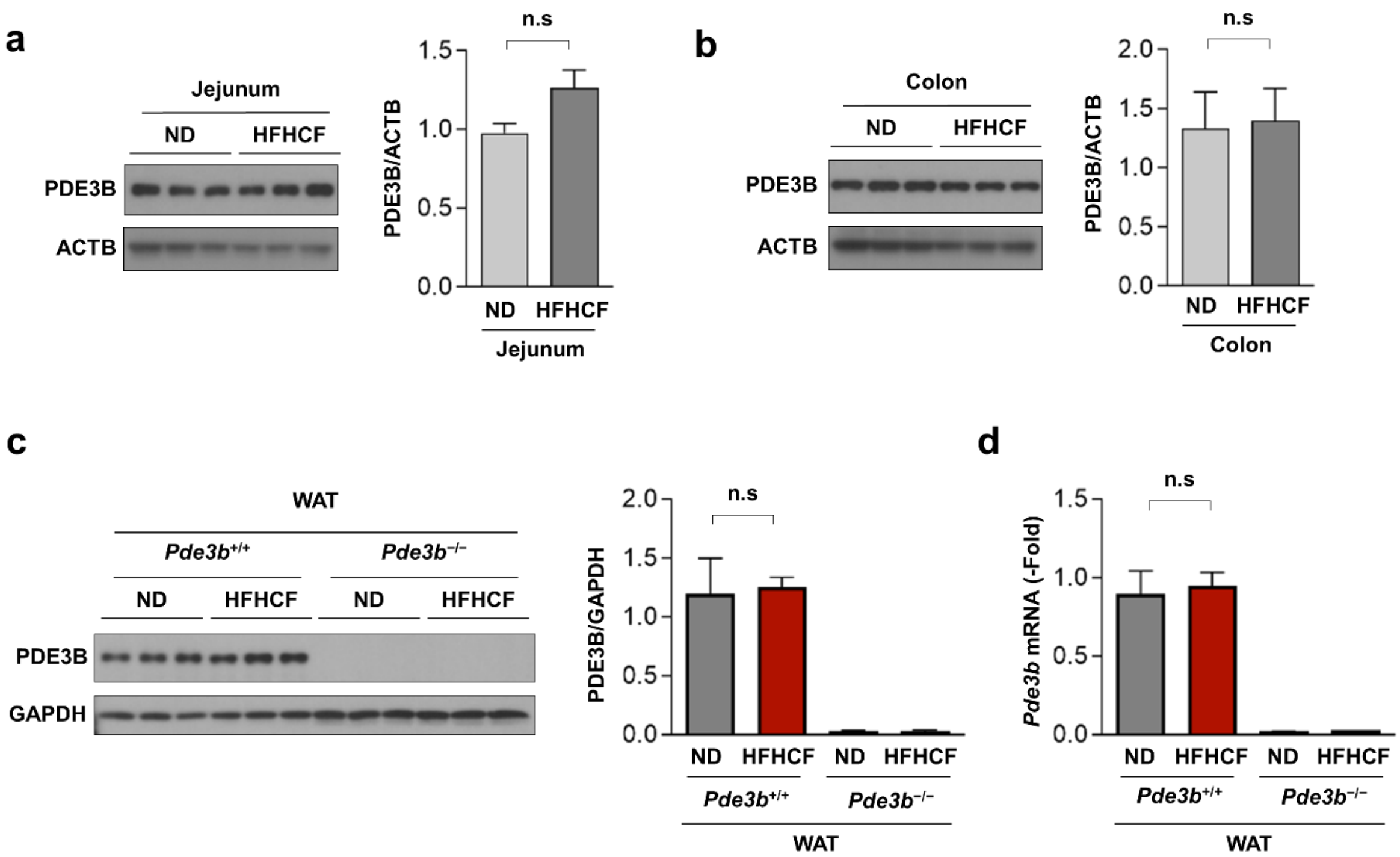

Supplementary Fig. 5. Ileal PDE3B expression is significantly reduced during MASH progression

(a, b) PDE3B protein expression in jejunum and colon tissues in 8 weeks HFHCF model. (c, d) Immunoblot and real-time qPCR analyses of PDE3B expression in epididymal white adipose tissue of ND- or HFHCF-fed mice (Cohort #6). Data are presented as mean ± SEM. n.s=non-significant. ND, normal diet; WAT, white adipose tissue; BAT, brown adipose tissue.

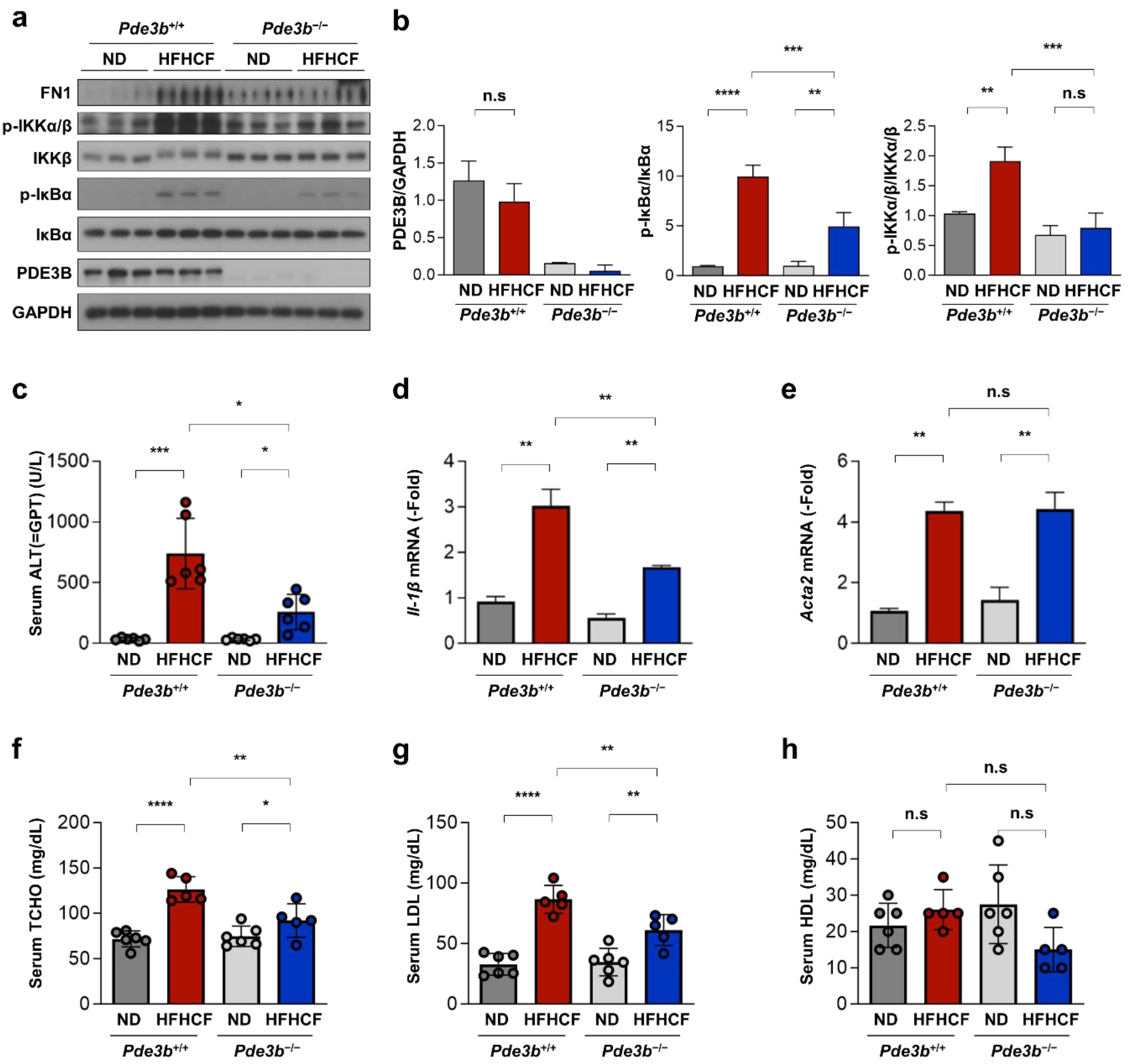

Supplementary Fig. 6. PDE3B promotes early hepatic inflammation and NF-κB activation in MASH progression

Pde3b$^{+/+}$ and *Pde3b*$^{-/-}$ mice in 2 weeks HFHCF model (Cohort #12). (a, b) Immunoblot analysis of fibrosis and inflammation-related proteins in liver tissues, with densitometric quantification. (c) Serum ALT levels measured in systemic blood samples. (d, e) Real-time qPCR analysis of indicated hepatic gene expression. (f–h) Serum total cholesterol, LDL, and HDL levels measured in systemic blood samples. Data are presented as mean ± SEM. *P < 0.0332; **P < 0.0021; ***P < 0.0002 and ****P < 0.0001. n.s=non-significant. ND, normal diet.

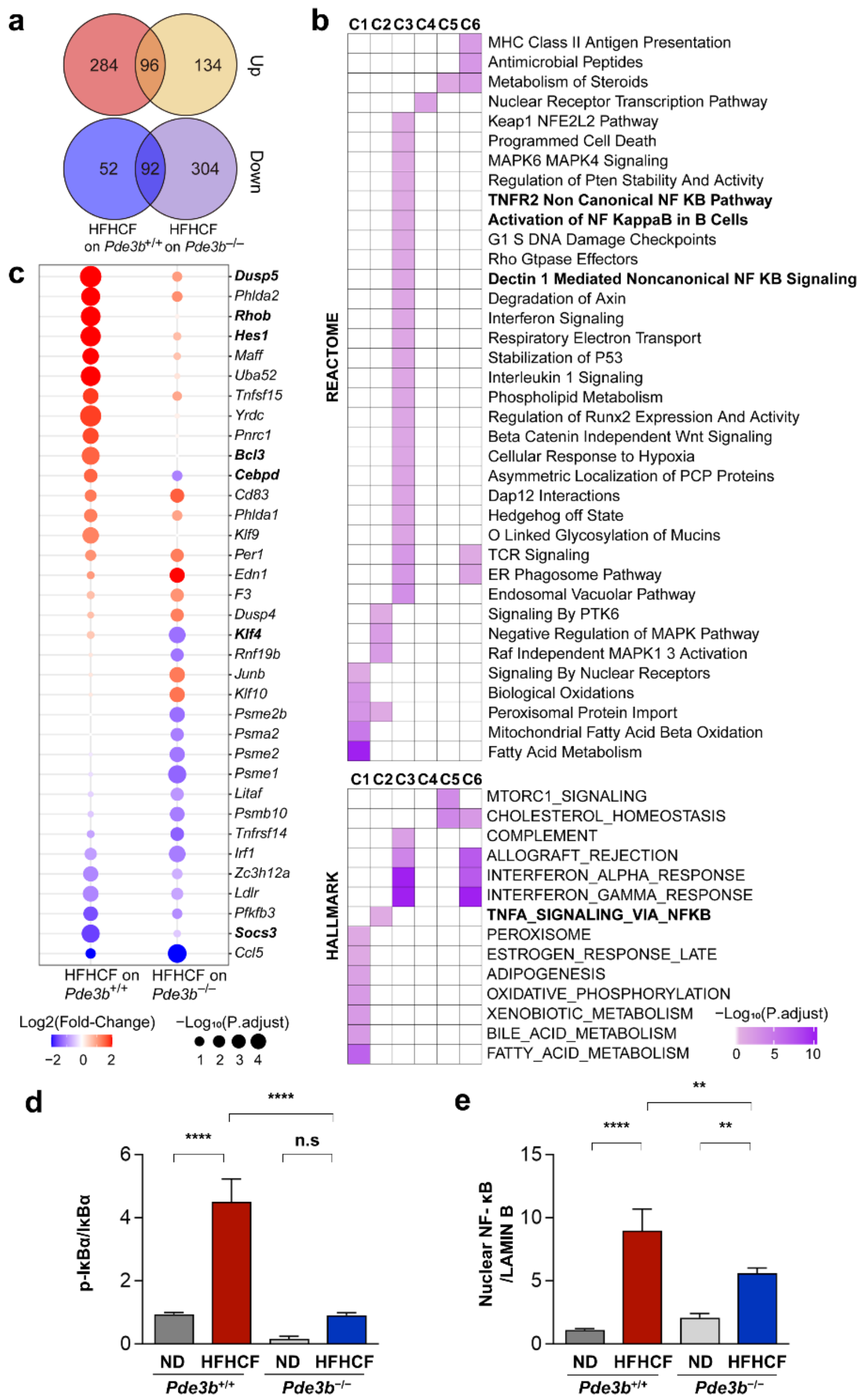

Supplementary Fig. 7. PDE3B regulates NF-κB signaling and pro-inflammatory gene expression in intestinal epithelial cells of MASH mice

Pde3b$^{+/+}$ and *Pde3b*$^{-/-}$ mice in 2 weeks HFHCF model, and IECs were isolated (Cohort #13). (a) Venn diagram showing overlapping and distinct DEGs between groups. (b) Gene set enrichment analysis (GSEA) of NF-κB signaling pathways using fgsea (v1.30.0). (c) DEG patterns in C2 and C3 groups from Figure 5C are highlighted. (d, e) The densitometric quantification of immunoblot from Figure 5f. Data are presented as mean ± SEM. $**P < 0.0021$ and $****P < 0.0001$. n.s=non-significant. IECs, intestinal epithelial cells.

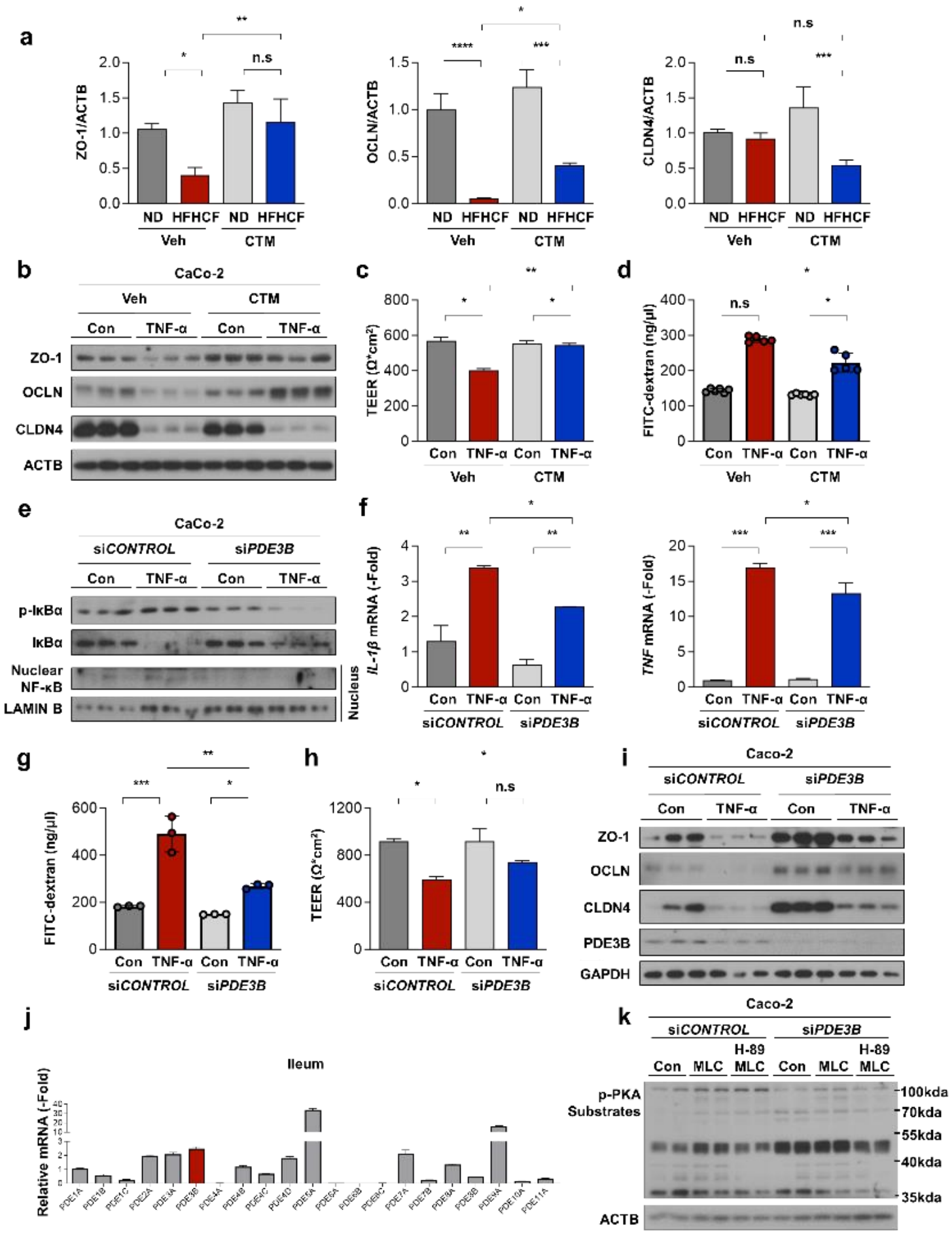

Supplementary Fig. 8. Pharmacological PDE3 inhibition and PDE3B depletion preserve epithelial barrier-associated readouts and suppress NF-κB activation.

(a) The densitometric quantification of immunoblot from Figure 6a. (b–d) Caco-2 cells were treated with TNF-α (50 ng/ml), with or without CTM (1 μM), for 48 h. (b) Immunoblotting analysis on pooled cell lysates with antibodies against indicated proteins. (c) Trans-epithelial electrical resistance (TEER) measurements using the EVOM2. (d) FITC-dextran fluorescence in transwell assays. (e–i) Caco-2 cells transfected with si*PDE3B* were treated with TNF-α (50 ng/ml) for 24 hours. (e) Immunoblot analysis of indicated proteins in Caco-2 cells. (f) Real-time qPCR analysis of pro-inflammatory genes in Caco-2 cells. (g) Supernatant FITC-dextran fluorescence measured in the bottom well of transwell assays. (h) TEER values were measured using EVOM2. (i) Immunoblot analysis of indicated proteins. (j) qPCR analysis of PDE isoforms in ileum tissue. (k) Caco-2 cells transfected with siPDE3B were treated with MLC, with or without H-89, for 24 h. Western blot analysis on cells with antibodies against indicated proteins. Data are presented as mean ± SEM. *P < 0.0332; **P < 0.0021; ***P < 0.0002; ****P < 0.0001. n.s=non-significant.

Supplementary Table. 1. The list of antibodies used for this study

Use	Antibodies	Source	Identifier
**Immune cell profiling**	Flow cytometry : Brilliant Violet 785™ anti-mouse CD4 Antibody	Biolegend	Cat. 100552
	Flow cytometry : Alexa Fluor® 700 anti-mouse/human CD44 Antibody	Biolegend	Cat. 103025
	Flow cytometry : BD OptiBuild™ BUV496 Hamster Anti-Mouse γδ T-Cell Receptor	BD	Cat. 750412
	Flow cytometry : Brilliant Violet 605™ anti-mouse TCR β chain Antibody	Biolegend	Cat. 109241
	Flow cytometry : BD Pharmingen™ PE-Cy™5 Rat Anti-Mouse CD8a	BD	Cat. 561094
	Flow cytometry : PerCP/Cyanine5.5 anti-mouse/human CD11b Antibody	Biolegend	Cat. 101227
	Flow cytometry: CD11c Monoclonal Antibody (N418), PerCP-Cyanine5.5	eBioscience	Cat. 45-0114-80
	Flow cytometry: CD14 Monoclonal Antibody (Sa2-8), PerCP-Cyanine5.5	eBioscience	Cat. 45-0141-80
	Flow cytometry: PerCP/Cyanine5.5 anti-mouse CD19 Antibody	Biolegend	Cat. 115533
	Flow cytometry: CD45R (B220) Monoclonal Antibody (RA3-6B2), PerCP-Cyanine5.5	eBioscience	Cat. 45-0452-80
	Flow cytometry: BD Horizon™ BUV395 Rat Anti-Mouse CD45	BD	Cat. 564279
	Flow cytometry: Pacific Blue™ anti-mouse CD11c Antibody	Biolegend	Cat. 117322
	Flow cytometry: BD Pharmingen™ FITC Rat Anti-CD11b	BD	Cat. 553310
	Flow cytometry: PE/Cyanine7 anti-mouse F4/80 Antibody	Biolegend	Cat. 123114
	Flow cytometry: Anti-Mo Ly-6G, APC	eBioscience	Cat. 17-9668-80
	Flow cytometry: APC-R700 Rat anti-mouse Siglec F	BD	Cat. 565183
	Flow cytometry: PerCP/Cyanine5.5 anti-mouse I-A/I-E	Biolegend	Cat. 107626
	Flow cytometry: Brilliant Violet 605™ anti-mouse CD64 (FcγRI)	Biolegend	Cat. 139323
	Flow cytometry: Brilliant Violet 785™ anti-mouse Ly-6C	Biolegend	Cat. 128041
	Flow cytometry : LIVE/DEAD™ Fixable Blue Dead Cell Stain Kit, for UV excitation	Invitrogen	Cat. L34961
	Flow cytometry : DAPI	Merck	Cat. D9542

**IHC**	Zo-1 polyclonal antibody	Invitrogen	Cat. 61-7300
	Vector Laboratories VECTASHIELD Antifade Mounting Medium With DAPI	Vector Laboratories	Cat. H-1200-10
	EpCAM Monoclonal Antibody (G8.8), APC	Invitrogen	Cat. 17-5791-82
	Goat anti-Rabbit IgG (H+L) Highly Cross-Adsorbed Secondary Antibody, Alexa Fluor™ 488	Invitrogen	Cat. A11034
**Immunoblotting**	PDE3B Monoclonal Antibody (SMCP3B)	Invitrogen	Cat. 14-1973-80
	beta Actin Antibody (C4)	Santa Cruz Biotechnology	Cat. SC-47778
	GAPDH Antibody (FL-335)	Santa Cruz Biotechnology	Cat. SC-25778
	Anti-alpha smooth muscle Actin antibody	Abcam	Cat. ab5694
	COL1A1 (E8F4L) XP ® Rabbit mAb	Cell Signaling Technology	Cat. 72026
	Anti-Fibronectin antibody	Abcam	Cat. ab2413
	Phospho-IκBα (Ser32) (14D4) Rabbit mAb	Cell Signaling Technology	Cat. 2859S
	IκBα Antibody	Cell Signaling Technology	Cat. 9242S
	NF-κB p65 (D14E12) XP ® Rabbit mAb	Cell Signaling Technology	Cat. 8242S
	Lamin B1 antibody	Proteintech	Cat. 12987-1-AP
	Anti-ZO1 tight junction protein antibody	Abcam	Cat. ab216880
	Anti-Occludin antibody [EPR20992]	Abcam	Cat. ab216327
	Claudin 4 Polyclonal Antibody (ZMD.306)	Invitrogen	Cat. 36-4800

Supplementary Table. 2. The list of chemicals and kits used for this study

	Name	Source	Identifier
**Chemicals Reagents Commercial assays**	CountBright™ Absolute Counting Beads	Invitrogen	Cat. C36950
	Collagenase D from Clostridium histolyticum	Sigma	Cat. 11088882001
	Collagenase from Clostridium histolyticum	Sigma	Cat. C5138
	Deoxyribonuclease I from bovine pancreas	Sigma	Cat. DN25
	Dispase	Worthington	Cat. LS02104
	Fetal bovine serum	HyClone	Cat. SH30919.03
	Percoll	Cytiva	Cat. 17089101
	EDTA, PH8	Biosesang	Cat. ER2002-050-80
	Dithiothreitol	Sigma	Cat. D9779
	HEPES	Welgene	Cat. BB001
	2-Mercaptoethanol	Gibco	Cat. 21985023
	L-glutamine	Welgene	Cat. LS002-01
	Penicillin-Streptomycin	Welgene	Cat. LS202-02
	Normal Goat Serum	Abcam	Cat. Ab7481
	Triton X-100	Biosesang	Cat. 9002-93-1
	TWEEN 20	Sigma	Cat. P1379
	Bovine Serum Albumin	Sigma	Cat. A9647
	Dulbecco's Phosphate Buffered Saline	HyClone	Cat. sh30028.02
	RPMI 1640 Medium	Welgene	Cat. sh30027.01
	HBSS without Ca, Mg	Welgene	Cat. LB 003-04
	HBSS (1X) (with Ca, Mg)	Welgene	Cat. LB 003-02
	TRIzol™ Reagent	Invitrogen	Cat. 15596026
	Aprotinin	Sigma	Cat. A3428
	DMSO	Sigma	Cat. D2650
	Fluorescein isothiocyanate-dextran	Sigma	Cat. 46944
	D-(-)-Fructose	Sigma	Cat. F01227
	Propylene Glycol	DUKSAN	Cat. 609
	Cilostamide	Enzo	Cat. BML-PD125-0025

	Hematoxylin solution	Sigma	Cat. 1.05175.2500
	Recombinant Human TNF-α	Pepro Tech	Cat. 300-01A
	Forskolin	Sigma	F3917
	H-89 dihydrochloride hydrate	Sigma	B1427
**Kits**	Enzy chrom triglyceride Assay KIT	BIOassay systems	Cat. ETGA-200
	Lipopolysaccharide ELISA Kit	Aviva system biology	Cat. OKEH02594
	Hydroxyproline Colorimetric Assay Kit	Biovision	Cat. K555
	Picosirius Red stain Kit	Polysciences	Cat. 24901-500
	Picosirius Red stain Kit	Abcam	Cat. ab150681
	GPT/ALT-P	FUJIFILM	Cat. 3250
	TCHO-P	FUJIFILM	Cat. 1450
	HDL-P	FUJIFILM	Cat. 2650

Supplementary Table. 3. The list of primers used for this study

Gene	Primer sequence	
**hPDE3B**	F	TTCAGGAGACCGTCGTTGC
	R	TGACACCATATTGCGAGCCTC
**hGAPDH**	F	GCGAGATCCCTCCAAAATCAA
	R	GTTCACACCCATGACGAACAT
**hIL-1β**	F	GGACAAGCTGAGGAAGATGC
	R	TCGTTATCCCATGTGTCGAA
**hTNF**	F	GTGACAAGCCTGTAGCCCAT
	R	TATCTCTCAGCTCCACGCCA
**mPDE3B**	F	GACCGTCGTTGCCTTGTATT
	R	CTCCATTTCCACCTCCAGAA
**mIl-1β**	F	AACCTGCTGGTGTGTGACGTTC
	R	CAGCACGAGGCTTTTTTGTTGT
**mCcl2**	F	TCTCTCTTCCTCCACCACCATG
	R	GCGTTAACTGCATCTGGCTGA
**mTnf**	F	GCCACCACGCTCTTCTG
	R	GGTGTGGGTGAGGAGCA
**mIl-6**	F	ACAACCACGGCCTTCCCTAC
	R	CACGATTTCCCAGAGAACATGTG
**mLy6c**	F	AGTCCTGTGTGCTCATTCTTC
	R	AGTCTCAATTGGCACTCCATAG
**mNos2**	F	CCAAGCCCTCACCTACTTCC
	R	CTCTGAGGGCTGACACAAGG
**mTrem2**	F	CACCATCACTCTGAAGAACCTC
	R	GCACCTCCACCAGTACTTTC
**mActa2**	F	CTGACAGAGGCACCACTGAA
	R	CATCTCCAGAGTCCAGCACA
**mCol1a1**	F	GAGCGGAGAGTACTGGATCG
	R	GCTTCTTTTCCTTGGGGTTC
**mTgfb1**	F	TTGCTTCAGCTCCACAGAGA
	R	GGGTCCCAGACAGAAGTTGG
**mPlin2**	F	GGGATCCCTGTCTACCAAGC
	R	AGATGTCGCCTGCCATCACC
**mDgat1**	F	GAGGCCTCTCTGCCCCTATG
	R	GCCCCTGGACAACACAGACT
**mSlc27a4**	F	AGTAAGCATGTGGCTTTGGGCAAG
	R	TTTGGCAGAAGATGGAGCAACAGC
**mCpt1a**	F	CACCAACGGGCTCATCTTCTA

	R	CAAAATGACCTAGCCTTCTATCGAA
**mAco1**	F	AGATTGGTAGAAATTGCTGCAAAA
	R	ACGCCACTTCCTTGCTCTTC
**mTjp1**	F	GGGAGGGTCAAATGAAGACA
	R	GGCATTCCTGCTGGTTACAT
**Genotyping**	**Primer sequence**	
**PDE3B**	O-11	ACACACCCCACAAAACACACCGTTGCACAGAACA
	E	CACGTTGCAGAAGCGGCAGAGGTGGAAGAAG
	N	AAAGCGCCTCCCCTACCCGGTAGAATTGACCTGCA

.

Supplementary Table. 4. The list of cell line and organism used for this study

	**Name**	**Source**	**Identifier**	
**Cell line**	Human Caco-2 cells (Passage no. p32-35)	KCLB	Cat. 30037.1	
	**Name**	**Source**	**Sex**	**Age**
**Mouse**	C57BL/6J	Japan SLC	male	8w
	Pde3b$^{+/+}$ and *Pde3b*$^{-/-}$	Developed by [6,8].	male	8w

Supplementary Table. 5. The list of software used for this study

**Software name**	**Manufacturer**	**Version**
GraphPad Prism	GraphPad software	Version 10.2.2
ImageJ	National Institutes of Health	Java8
Adobe Illustrator	Adobe	2023
R	R Foundation	4.4.2
Rstudio Desktop	Posit, PBC	2024.12.0+467
Mobaxterm	Mobatek	24.4

Supplementary Table. 6. The list of deposited data used for this study

Name of repository	Identifier	Link
RNA-seq profile of ileum IECs from Pde3b wild-type and knockout (Pde3b−/−) mice after 2 weeks of either a normal diet or a HFHCF diet	This paper	GSE285516
Human liver biopsy of different phases from control to NASH	[9]	GSE48452
Spatial proteogenomics reveals distinct and evolutionarily-conserved hepatic macrophage niches (single cells)	[10]	GSE192740

Supplementary Table. 7. Animal cohort allocation and sample sizes

Cohort No.	Set	Group				Note
	MASH	Normal		MASH		
**1**	CDAHFD	4		5		
**2**	MCD	3		5		
**3**	patient	3		3		
	**genetic**	*Pde3b*$^{+/+}$ ND	*Pde3b*$^{+/+}$ HFHCF	*Pde3b*$^{-/-}$ ND	*Pde3b*$^{-/-}$ HFHCF	
**4**	set 1 (8w)	3	4	4	4	
**5**	set 2 (8w)	5	4	5	5	
**6**	set 3 (8w)	6	6	5	5	
**7**	set 4 (8w)	6	7	6	6	Flow cytometry (liver)
**8**	set 5 (8w)	3	3	3	3	Flow cytometry (Ileum)
**9**	set 6 (8w)	5	4	5	4	FITC-dextran
**10**	set 7 (8w)	3	3	3	3	portal vein
	Total (per arm)	31	31	31	30	
**11**	set 1 (4w)	5	5	5	5	
**12**	set 1 (2w)	6	6	6	6	
**13**	set 2 (2w)	3	4	4	4	IEC mRNA seq
**14**	set 3 (2w)	3	3	3	4	FITC-dextran
**15**	set 4 (2w)	4	5	3	6	portal vein
	Total (per arm)	16	18	16	20	
	**pharmacol**	Veh ND	Veh HFHCF	CTM ND	CTM HFHCF	
**16**	set 1 (8w)	5	5	5	5	
**17**	set 2 (8w)	5	5	5	5	
**18**	Set 3 (8w)	4	4	4	4	FITC-dextran
	Total (per arm)	14	14	14	14	
		Pde3b$^{+/+}$ HFHCF-Veh	*Pde3b*$^{+/+}$ HFHCF-CTM	*Pde3b*$^{-/-}$ HFHCF-Veh	*Pde3b*$^{-/-}$ HFHCF-CTM	
**19**	set 1 (8w)	10	10	10	10	

Reference

1. Lee, D.H. *et al.* Fecal microbiota transplantation improves hepatic fibro-inflammation via regulating oxidative stress in experimental NASH. *Dig Liver Dis*. **55**, 1521-1532 (2023).
2. Whitaker, R.M. Wills, L.P. Stallons, L.J. & Schnellmann, R.G. cGMP-selective phosphodiesterase inhibitors stimulate mitochondrial biogenesis and promote recovery from acute kidney injury. *J Pharmacol Exp Ther*. **347**, 626-634 (2013).
3. Sancar, G. *et al.* FGF1 and insulin control lipolysis by convergent pathways. *Cell Metab*. **34**, 171-183 e176 (2022).
4. Haas, J.T. *et al.* Hepatic insulin signaling is required for obesity-dependent expression of SREBP-1c mRNA but not for feeding-dependent expression. *Cell Metab*. **15**, 873-884 (2012).
5. Repa, J.J. *et al.* Regulation of mouse sterol regulatory element-binding protein-1c gene (SREBP-1c) by oxysterol receptors, LXRalpha and LXRbeta. *Genes Dev*. **14**, 2819-2830 (2000).
6. Chung, Y.W. *et al.* Targeted disruption of PDE3B, but not PDE3A, protects murine heart from ischemia/reperfusion injury. *Proc Natl Acad Sci U S A*. **112**, E2253-2262 (2015).
7. Chung, Y.W. *et al.* White to beige conversion in PDE3B KO adipose tissue through activation of AMPK signaling and mitochondrial function. *Sci Rep*. **7**, 40445 (2017).
8. Guirguis, E. *et al.* A role for phosphodiesterase 3B in acquisition of brown fat characteristics by white adipose tissue in male mice. *Endocrinology*. **154**, 3152-3167 (2013).
9. Ahrens, M. *et al.* DNA methylation analysis in nonalcoholic fatty liver disease suggests distinct disease-specific and remodeling signatures after bariatric surgery. *Cell Metab*. **18**, 296-302 (2013).
10. Guilliams, M. *et al.* Spatial proteogenomics reveals distinct and evolutionarily conserved hepatic macrophage niches. *Cell*. **185**, 379-396 e338 (2022).
